# Plasticity and therapeutic potential of cAMP and cGMP-specific phosphodiesterases in *Toxoplasma gondii*

**DOI:** 10.1016/j.csbj.2022.09.022

**Published:** 2022-09-24

**Authors:** Kim Chi Vo, Liberta Ruga, Olympia Ekaterini Psathaki, Rico Franzkoch, Ute Distler, Stefan Tenzer, Michael Hensel, Peter Hegemann, Nishith Gupta

**Affiliations:** a Department of Molecular Parasitology, Institute of Biology, Faculty of Life Sciences, Humboldt University, Berlin, Germany; b University of Osnabrück, Center of Cellular Nanoanalytics (CellNanOs), Integrated Bioimaging Faciltiy (iBiOs), Germany; c Institute of Immunology, University Medical Center of the Johannes-Gutenberg University Mainz, Mainz, Germany; d Department of Biological Sciences, Birla Institute of Technology and Science, Pilani (BITS-P), Hyderabad, India

**Keywords:** 3′IT, 3′-insertional tagging, COS, crossover sequence, CRISPR, clustered regularly interspaced short palindromic repeats, DHFR-TS, dihydrofolate reductase – thymidylate synthase, HFF, human foreskin fibroblast, HXGPRT, hypoxanthine-xanthine-guanine phosphoribosyl transferase, IMC, inner membrane complex, MoI, multiplicity of infection, PDE, phosphodiesterase, PKA, protein kinase A, PKG, protein kinase G, PM, plasma membrane, S. C., selection cassette, smHA, spaghetti monster-HA, *sg*RNA, single guide RNA, TEM, transmission electron microscopy, Apicomplexa, Phosphodiesterase, cAMP & cGMP signaling, Lytic cycle, Tachyzoite

## Abstract

*Toxoplasma gondii* is a common zoonotic protozoan pathogen adapted to intracellular parasitism in many host cells of diverse organisms. Our previous work has identified 18 cyclic nucleotide phosphodiesterase (PDE) proteins encoded by the parasite genome, of which 11 are expressed during the lytic cycle of its acutely-infectious tachyzoite stage in human cells. Here, we show that ten of these enzymes are promiscuous dual-specific phosphodiesterases, hydrolyzing cAMP and cGMP. *Tg*PDE1 and *Tg*PDE9, with a K_m_ of 18 μM and 31 μM, respectively, are primed to hydrolyze cGMP, whereas *Tg*PDE2 is highly specific to cAMP (K_m_, 14 μM). Immuno-electron microscopy revealed various subcellular distributions of *Tg*PDE1, 2, and 9, including in the inner membrane complex, apical pole, plasma membrane, cytosol, dense granule, and rhoptry, indicating spatial control of signaling within tachyzoites. Notably, despite shared apical location and dual-catalysis, *Tg*PDE8 and *Tg*PDE9 are fully dispensable for the lytic cycle and show no functional redundancy. In contrast, *Tg*PDE1 and *Tg*PDE2 are individually required for optimal growth, and their collective loss is lethal to the parasite. *In vitro* phenotyping of these mutants revealed the roles of *Tg*PDE1 and *Tg*PDE2 in proliferation, gliding motility, invasion and egress of tachyzoites. Moreover, our enzyme inhibition assays in conjunction with chemogenetic phenotyping underpin *Tg*PDE1 as a target of commonly-used PDE inhibitors, BIPPO and zaprinast. Finally, we identified a retinue of *Tg*PDE1 and *Tg*PDE2-interacting kinases and phosphatases, possibly regulating the enzymatic activity. In conclusion, our datasets on the catalytic function, physiological relevance, subcellular localization and drug inhibition of key phosphodiesterases highlight the previously-unanticipated plasticity and therapeutic potential of cyclic nucleotide signaling in *T. gondii*.

## Graphical abstract

**Figure:**
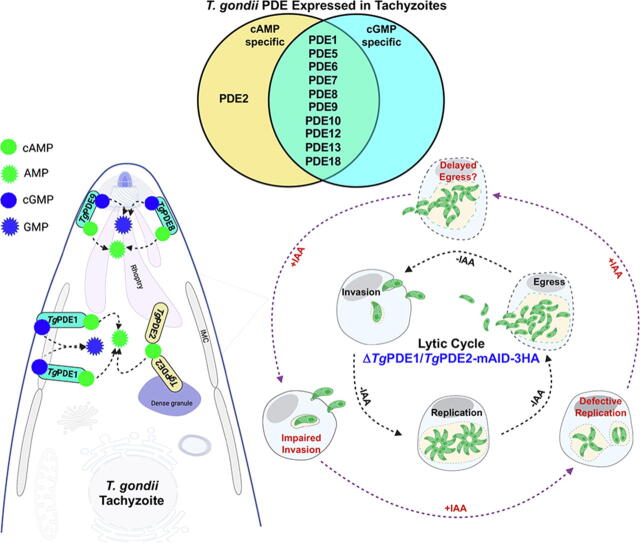

## 1 Introduction

Cyclic nucleotide signaling in apicomplexan parasites has been an active area of research in the last decade. Its phylogenetic divergence, *modus operandi,* and functional repurposing to enable the specialized lifecycle events in this class of clinically-relevant pathogens have attracted the most attention. *Toxoplasma* and *Plasmodium* are the two standard parasite models deployed to study apicomplexan biology, including cAMP and cGMP signaling. *T. gondii* – the only known species of *Toxoplasma,* is well known for its prominent ability to infect and reproduce in several warmblood organisms without geographic constraints [1]. The parasite undergoes asexual and sexual growth switching between multiple infectious stages, and exhibits exceptional promiscuity and metabolic plasticity, which underlie its widespread infection, inter-host transmission, reproduction, persistence, and pathogenesis. It is, therefore, imperative to examine the molecular mechanisms and determinants of infection and develop efficient anti-parasitic treatment strategies.

This work focuses on the tachyzoite stage of *T. gondii* responsible for the acute infection (tissue necrosis by recurrent lytic cycles). Tachyzoites can infect a broad range of nucleated host cells in humans and animals. The lytic cycle comprises several steps, such as gliding motility, invasion, proliferation, and egress [2]. Besides other known factors, protein kinase-dependent on cAMP (PKA) and protein kinase-dependent on cGMP (PKG) serve as the prime regulators of the lytic cycle events. Cyclic GMP signaling, for example, governs the calcium-dependent micronemal exocytosis needed for the motility-driven invasion and egress by the parasite. It is initiated by an exclusive guanylate cyclase fused to a P4-type ATPase (ATPase_P_-GC), and mediated by PKG [3], [4], [5], [6], [7]. On the other hand, cAMP signaling, facilitated by adenylate cyclase and PKA proteins, has been suggested as a negative regulator of PKG and associated Ca^2+^ homeostasis/signaling [8], [9]. In addition, cAMP is known to regulate the acute-chronic stage differentiation in *T. gondii* [10], [11], [12].

While the actuation of cAMP and cGMP signaling is relatively well studied, their counter-regulation remains poorly understood in *T. gondii*. Cyclic nucleotide phosphodiesterase (PDE) enzymes are crucial for the spatiotemporal repression of signaling cascades. *T. gondii* harbors a remarkably expanded repertoire of phosphodiesterases, and many of them are phylogenetically divergent from their human counterparts [13], [14]. Our earlier work has revealed that of the 18 PDEs present in *T. gondii*, a panel of 11 proteins is expressed at different subcellular locations in tachyzoites [14], which was endorsed in a recent study [15]. We also demonstrated *Tg*PDE8 and *Tg*PDE9 as dual-specific (hydrolyzing cAMP and cGMP) enzymes and determined that the latter is not essential for the lytic cycle. *Tg*PDE1 and *Tg*PDE2, on the other hand, are presumed to be crucial for the lytic cycle based on conditional mutagenesis in tachyzoites [15]. Nonetheless, the substrate specificity, physiological relevance, functional redundancy and therapeutic potential of these and other PDEs remain severely underexplored in *T. gondii*, which inspired us to perform this study.

## 2 Results

### 2.1 *Tg*PDE2 is cAMP-specific, while other PDEs in tachyzoites can hydrolyze cAMP and cGMP

**Fig. 1:**
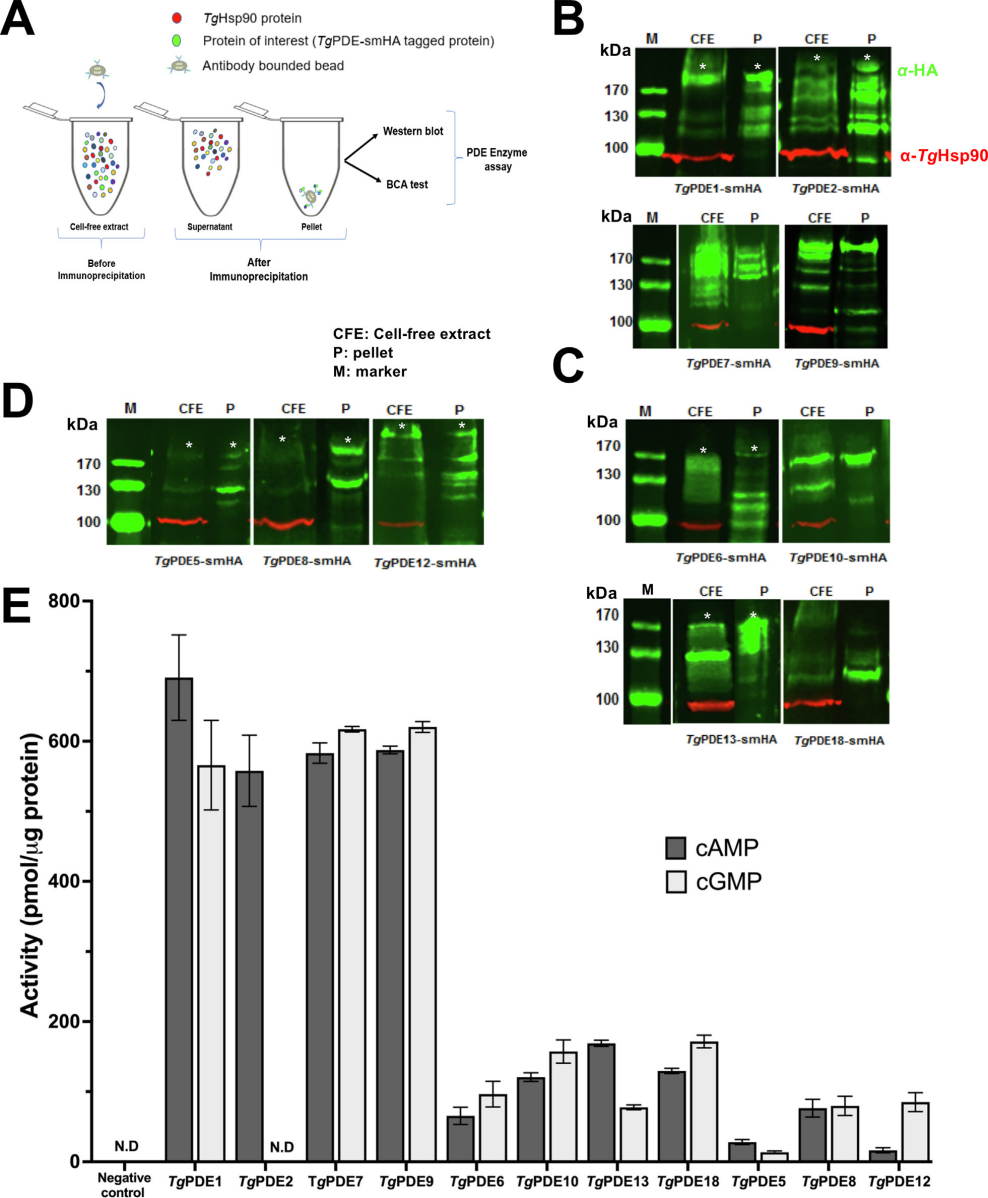
Catalytic activity of the native phosphodiesterases present in tachyzoites of *T. gondii*. **(A)** Scheme for the α-HA agarose bead-based immunoprecipitation to enrich the smHA-tagged PDE proteins from the cell lysate of transgenic parasites. The parental strain (*RH*Δ*ku80*Δ*hxgprt*) was used as a negative control. **(B-D)** Immunoblots confirming adequate precipitation of PDE-smHA proteins. Samples from *panel A* were probed with the mouse α-HA (green) and rabbit α-*Tg*Hsp90 (red) antibodies. Note the presence of *Tg*Hsp90 (a cytosolic marker) in cell-free extract (CFE) and its absence in the immunoprecipitated pellet (P). Asterisks, if shown, mark the predicted size of smHA-tagged PDEs. **(E)** Phosphodiesterase activity of PDE-smHA proteins with cAMP and cGMP. The colorimetric enzyme assays were set up using 6 μg of PDE samples (*Tg*PDE5, 10 μg) and 200 μM substrate (1 h, 37 °C). The control reactions run alongside lacked the substrate or enzyme. The substrate-free enzyme-only reaction was subtracted from samples to quantify the PDE activity (normalized to the protein amount). The negative controls indicate the precipitated protein of the parental strain (N.D., not detectable). The data show the mean ± SE (n = 3–4 assays).

We performed the colorimetric enzyme assays using enriched preparation of the native PDEs. Transgenic tachyzoites encoding smHA-tagged proteins under the control of respective promoters were deployed to isolate the 11 PDEs expressed during the lytic cycle. As shown (Fig. 1A), these proteins were immunoprecipitated from the parasite extract, and PDE-enriched samples were tested by immunoblot and enzyme assays (Fig. 1B-E). Similar to the earlier work [14], we observed a protein band of the predicted size for most PDEs besides some proteolytic products (Fig. 1B-D). Except for *Tg*PDE2, all other enzymes hydrolyzed both substrates, *i.e.*, cAMP and cGMP (Fig. 1E). *Tg*PDE2 degraded only cAMP with no evident activity for cGMP even at a much higher amount of the protein (20 µg) and substrate (200 μM). *Tg*PDE2, with a catalytic rate of > 0.5 nmol/µg protein, was also among the most active enzymes. *Tg*PDE1, *Tg*PDE7 and *Tg*PDE9 were similarly efficient in hydrolyzing cGMP and cAMP (Fig. 1E).

The second cohort of enzymes with relatively moderate activity (0.1–0.2 nmol/µg protein) included *Tg*PDE6, *Tg*PDE10, *Tg*PDE13, and *Tg*PDE18 (Fig. 1E). Except for *Tg*PDE13, which was twice as functional with cAMP than cGMP, the other three PDEs displayed similar rates of catalysis with both substrates. The third and last set of phosphodiesterases comprising *Tg*PDE5, *Tg*PDE8, and *Tg*PDE12 was much less active (<0.1 nmol/µg protein), correlating with their weak expression in tachyzoites (Fig. 1D). *Tg*PDE5 appeared at least twice more active with cAMP than cGMP. Conversely, *Tg*PDE12 hydrolyzed cGMP at a 5x higher rate than cAMP. Collectively, these assays revealed the catalytic specificity of PDEs expressed in tachyzoites and suggested a significant functional redundancy in the counter-regulation of cyclic nucleotide signaling.

### 2.2 Catalytic kinetics of *Tg*PDE1, *Tg*PDE2, *Tg*PDE7 and *Tg*PDE9 with cAMP and/or cGMP

**Fig. 2:**
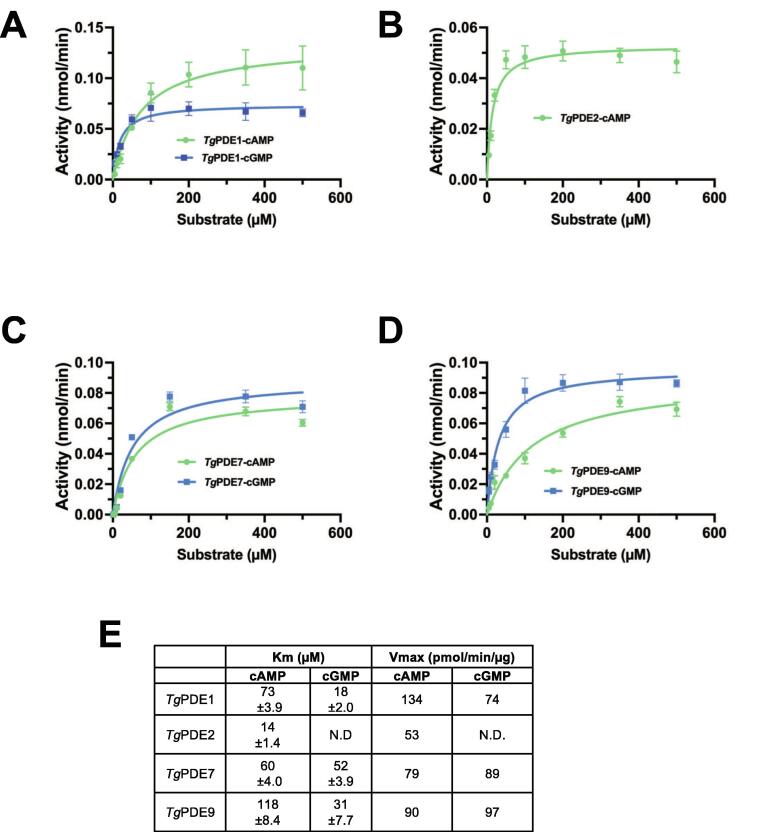
Substrate dependence of *Tg*PDE1, *Tg*PDE2, *Tg*PDE7 and *Tg*PDE9 enzymes. **(A-D)** Michaelis-Menten plots of PDE proteins at different concentrations of cAMP or cGMP. The enzyme assays were executed under standard conditions using immunoprecipitated PDE-smHA samples (see Fig. 1A). For the protein and time dependence of the PDE activity, refer to Fig. S1. **(E)** The K_m_ and V_max_ values of PDEs. The kinetic parameters were calculated based on the data presented in *panels A-D* (mean ± SE from 3 to 5 assays).

Given the high enzymatic activity, distinct subcellular locations and the yield of enriched proteins, we focused on the substrate kinetics of *Tg*PDE1, *Tg*PDE2, *Tg*PDE7 and *Tg*PDE9 (Fig. 2). At first, the enzyme and time dependence of each PDE were assessed under saturating amount of cAMP or cGMP (Fig. S1). Knowing the linearity of individual reactions, we tested the catalytic activity with 1–500 µM of substrates to calculate the K_m_ and V_max_ values. All four PDEs displayed the typical Michaelis-Menten kinetics showing a dependence of their catalysis on the substrate concentration (Fig. 2A-E). *Tg*PDE1, with a K_m_ of 73 µM for cAMP and 18 µM for cGMP, had a 4-fold higher affinity for the latter (Fig. 2A). *Tg*PDE2, exhibiting the lowest K_m_ (14 µM) among all, was functional only with cAMP (Fig. 2B). *Tg*PDE7 with the K_m_ values of 60 µM and 52 µM for cAMP and cGMP, respectively, displayed a similar affinity for both cyclic nucleotides (Fig. 2C). On the other hand, the hydrolytic activity of *Tg*PDE9 for cAMP (K_m_, 118 µM) and cGMP (K_m_, 31 µM) was analogous to *Tg*PDE1 (Fig. 2D). As elaborated below, the kinetic parameters of indicated PDEs enabled the interpretation of our mutagenesis and phenotyping datasets besides elucidating their pharmacological relevance in the context of known inhibitors.

### 2.3 Effect of PDE inhibitors on the parasite phosphodiesterases

**Fig. 3:**
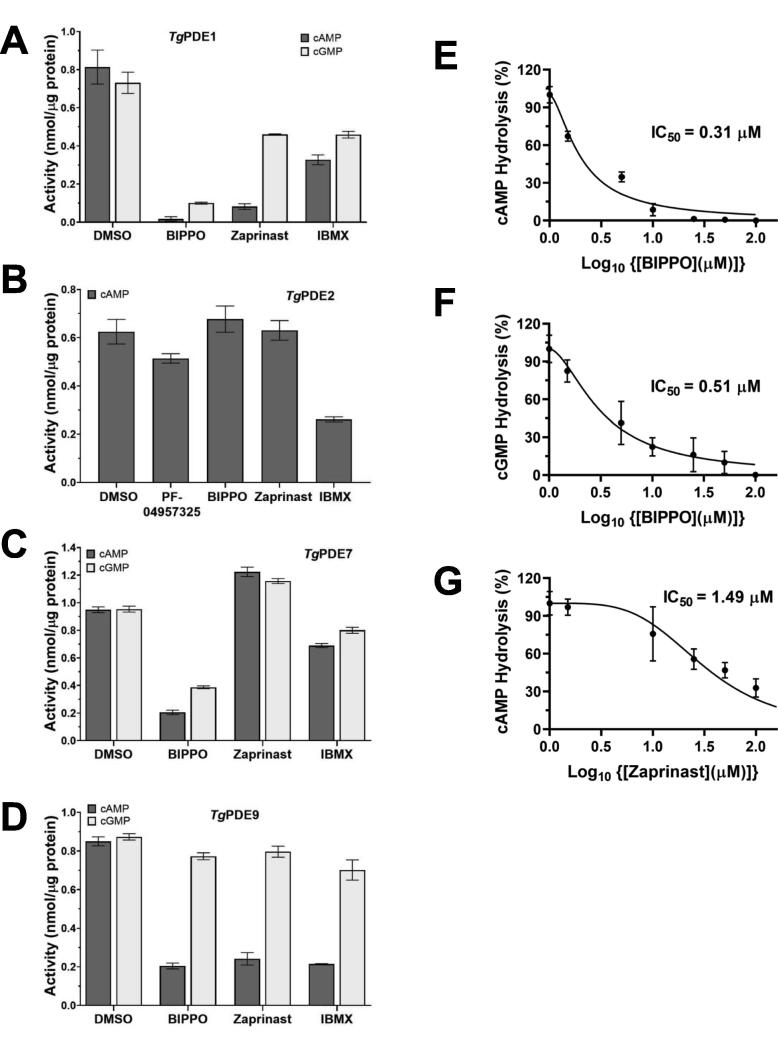
Effect of inhibitors on cAMP and cGMP hydrolysis by *Tg*PDE1, *Tg*PDE2, *Tg*PDE7, and *Tg*PDE9. **(A-D)** Enzymatic activity of PDEs in the presence of BIPPO (100 µM), zaprinast (300 µM), PF-04957325 (50 µM), and IBMX (100 µM). The assay was performed under standardized conditions at 37 °C. DMSO was included as solvent control. Note the inhibition of *Tg*PDE1 but not of *Tg*PDE2 by BIPPO and zaprinast. **(E-G)** Inhibition kinetics of cAMP or cGMP hydrolysis by *Tg*PDE1. The IC_50_ values for each pair of substrate-inhibitor were calculated by the log transform of the drug concentration *versus* the normalized enzyme activity. Graphs in *panels A-G* depict the mean ± SE from 3 to 5 experiments.

In extended enzyme assays, we examined the inhibition kinetics of the *Tg*PDE1, *Tg*PDE2, *Tg*PDE7, and *Tg*PDE9 preparations by BIPPO, zaprinast, IBMX, and PF-04957325 (Fig. 3). BIPPO and zaprinast are assumed to inhibit cGMP hydrolysis and widely deployed to induce the PKG-dependent egress in tachyzoites [7], [8], [13], [16], [17], whereas IBMX (3-isobutyl-1-methylxanthine) is a nonselective drug inhibiting human phosphodiesterases [18], and PF-04957325 specifically blocks the cAMP-specific human PDE8 [19], [20]. In our inhibition assays involving the parasite PDEs (Fig. 3A), hydrolysis of both cyclic nucleotides by *Tg*PDE1 was strongly inhibited (>90 %) in the presence of BIPPO. However, zaprinast was more potent in blocking cAMP degradation (>80 %) compared to cGMP (∼40 %). The effect of IBMX on *Tg*PDE1 was moderate. Notably, hydrolysis of cAMP by *Tg*PDE2 was sensitive to partial inhibition by IBMX but to none of the other drugs (Fig. 3B). On the other hand, *Tg*PDE7-mediated catalysis of cAMP and cGMP were reduced by > 70 % upon inclusion of only BIPPO (Fig. 3C). Consistent with its differential substrate affinity (Fig. 2E), the activity of *Tg*PDE9 for cAMP was inhibited (>75 %) by BIPPO, zaprinast as well as IBMX, but its catalytic action on cGMP remained largely unaffected (Fig. 3D).

A remarkably strong effect of BIPPO and zaprinast on *Tg*PDE1 prompted us to perform the inhibition kinetics using different concentrations of inhibitors. The IC**_50_** of BIPPO for the cAMP and cGMP hydrolysis by *Tg*PDE1 was 0.31 µM (Fig. 3E) and 0.51 µM (Fig. 3F), respectively. In contrast, zaprinast exhibited an approximately 5x higher IC_50_ value (1.49 µM) for cAMP (Fig. 3G). These data advocate that commonly-used inducers of parasite egress can inhibit cAMP as well as cGMP hydrolysis and indicate *Tg*PDE1 as the primary target of these drugs.

### 2.4 *Tg*PDE7, *Tg*PDE8, and *Tg*PDE9 are expendable during the lytic cycle

Our preceding work has shown that tachyzoite can survive the genetic deletion of *Tg*PDE9 with no apparent defect in the lytic cycle [14]. Herein, we tested the physiological importance and functional redundancy of other designated PDEs by CRISPR/Cas9-aided mutagenesis (Figs. S2-S4A). The gene-specific knockout constructs with 5′ and 3′ homology arms flanking a DHFR-TS selection cassette were transfected into respective progenitor parasite strains expressing smHA-tagged PDEs. Transgenic tachyzoites were selected by pyrimethamine, and mutants were isolated by limiting dilution. The genomic screening of clonal mutants by PCR revealed the occurrence of 5′ and 3′-crossovers, confirming a successful replacement of the *Tg*PDE7, *Tg*PDE8, and *Tg*PDE9 *loci* by the selection marker (Figs. S2-S4B). The loss of PDE expression in transgenic parasites was tested by immunofluorescence and immunoblot methods (Figs. S2-S4C). Unlike the matching progenitor strains, no HA signal was observed in the mutants. Plaque assays (Figs. S2-S4D), representing the periodic lytic cycles and thus the overall fitness of tachyzoites, disclosed a normal growth in the parental and progenitor strains, as expected. Surprisingly, however, none of the three mutants (Δ*Tg*PDE7, Δ*Tg*PDE8, or Δ*Tg*PDE9) exhibited a growth defect, as deduced by the number and size of plaques.

**Fig. 4:**
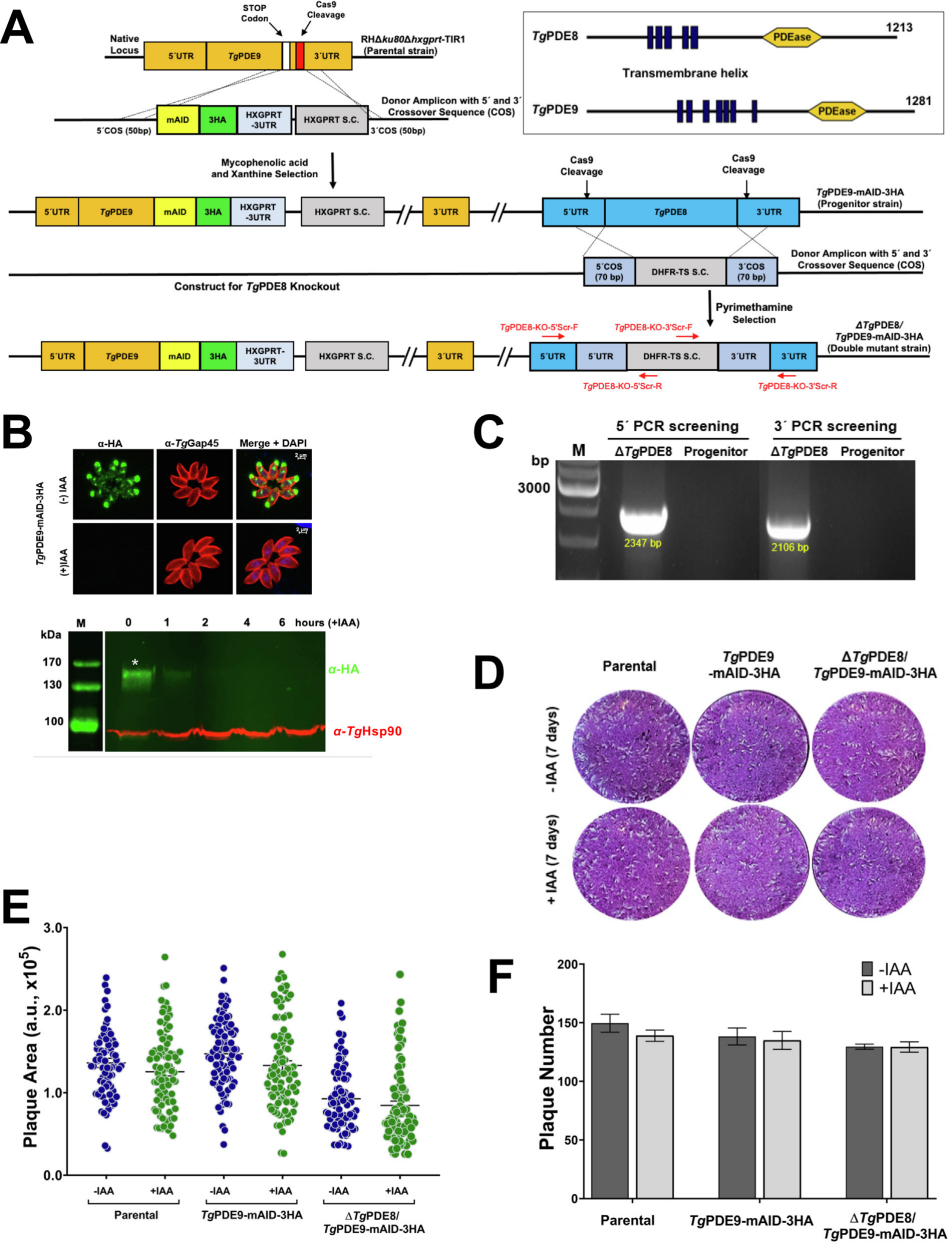
*Tg*PDE8 and *Tg*PDE9 are dispensable individually and do not complement each other during the lytic cycle. **(A)** The primary structures of *Tg*PDE8 and *Tg*PDE9 as well as schematics for making a double mutant (Δ*Tg*PDE8 in *Tg*PDE9-mAID-3HA strain) by CRISPR/Cas9-assisted homologous crossover. In the first step, an auxin-regulated mutant of *Tg*PDE9 was generated, which also served as a progenitor strain for deleting the *TgPDE8* gene. **(B)** Immunofluorescence and immunoblot showing the dependence of *Tg*PDE9 expression on indole-3-acetic acid (IAA)**.** For immunofluorescence, parasites were cultured for 24 h without or with IAA before staining the HA-tag (green) and *Tg*Gap45 (inner membrane complex, red). Immunoblots were generated after IAA treatment for the indicated periods. *Tg*Hsp90 served as a loading control. **(C)** Genomic PCR with recombination-specific primers, confirming the 5′ and 3′ recombination events at the *TgPDE8* locus in the double mutant. For the primer binding sites, see *panel A*. **(D-F)** Plaque assays to test the growth of the *Tg*PDE9-mAID-3HA and Δ*Tg*PDE8/*Tg*PDE9-mAID-3HA mutants in comparison to the *RH*Δ*ku80*Δ*hxgprt*-TIR1 (parental) strain. The white speckles on a blue background signify the parasite plaques formed in a host-cell monolayer. Graphs show the area **(E)** and number **(F)** of plaques, manifesting the fitness of tachyzoites in the absence or presence of IAA. For *panel E*, the size of 150–200 plaques of each strain from 3 replicates was scored in arbitrary units (a. u.).

We subsequently generated a double mutant to investigate the possible redundancy between the two apically-located dual-specific PDEs, *i.e.*, *Tg*PDE8 and *Tg*PDE9 (Fig. 4). First, a conditional *Tg*PDE9 mutant was made by 3′-genomic tagging with a mini auxin-inducible degron (mAID) using the HXGPRT selection marker (Fig. 4A). The mAID system enables maintaining viable parasites in the absence of indole-3-acetic acid (IAA, a type of auxin) if the gene is essential [4]. A plasmid expressing Cas9 nuclease and gene-specific *sg*RNA targeting the *Tg*PDE9-3′UTR was transfected with a donor amplicon (5′ and 3′-homology arms flanking mAID-3HA and HXGPRT selection cassette) into tachyzoites. Immunostaining confirmed the apical localization and protein integrity (Fig. 4B). The HA signal was not detectable within 1 h of auxin treatment, ratifying a fast and efficient conditional knockdown of *Tg*PDE9 in tachyzoites (see immunoblot). In the second step, we deleted *Tg*PDE8 in the *Tg*PDE9-mAID-3HA strain by double homologous crossover and pyrimethamine selection (Fig. 4A). The eventual mutant was screened by genomic PCR (Fig. 4C). Compared to the parental parasites, neither the *Tg*PDE9-mAID-3HA nor Δ*Tg*PDE8/*Tg*PDE9-mAID-3HA strain was affected in plaque assays irrespective of auxin supplementation (Fig. 4D-F), suggesting no apparent functional overlap between the two enzymes during the lytic cycle of *T. gondii*.

### 2.5 *Tg*PDE1 and *Tg*PDE2 are partly redundant but individually needed for the parasite growth

**Fig. 5:**
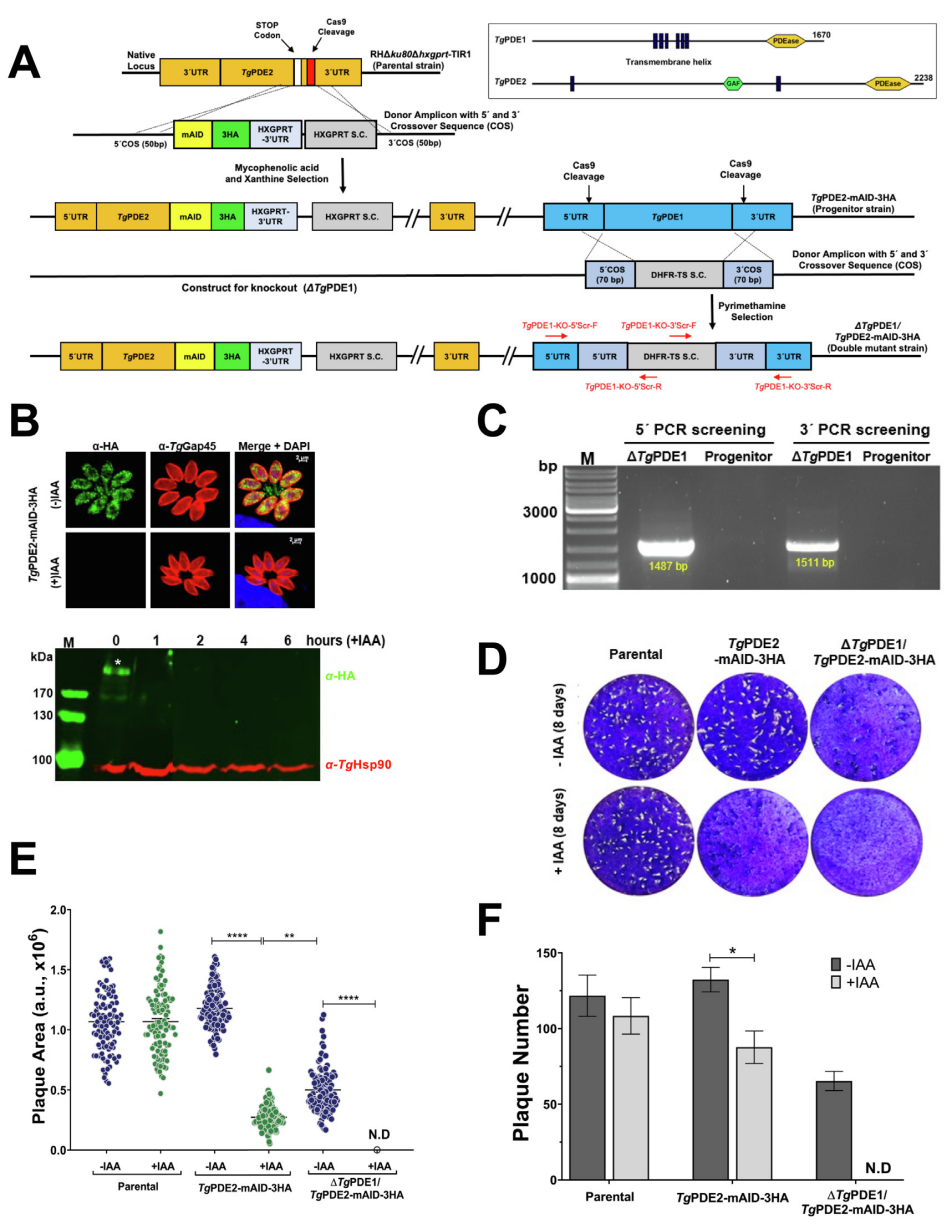
*Tg*PDE1 and *Tg*PDE2 are required for the lytic cycle, and the loss of both proteins is lethal to tachyzoites. **(A)** The primary structures of *Tg*PDE1 and *Tg*PDE2, as well as the graphics for making the Δ*Tg*PDE1/*Tg*PDE2-mAID-3HA strain by CRISPR/Cas9-assisted genetic manipulation. As illustrated, first, a conditional (auxin-regulated) mutant of *Tg*PDE2 was constructed, which was deployed as a progenitor strain for the *Tg*PDE1 deletion. **(B)** Immunofluorescence and immunoblot images depicting a depletion of *Tg*PDE2 by IAA**.** Tachyzoites were cultured for 24 h (+/-IAA) before anti-HA and anti-*Tg*Gap45 immunostaining and microscopy. For immunoblots, parasites were treated with IAA, as shown (*Tg*Hsp90, loading control). **(C)** Genomic screening of the double mutant using 5′ and 3′-crossover-specific primers confirming the deletion of *Tg*PDE1. **(D-F)** Plaque assays to test the relative growth of the *Tg*PDE2-mAID-3HA, Δ*Tg*PDE1/*Tg*PDE2-mAID-3HA, and *RH*Δ*ku80*Δ*hxgprt*-TIR1 (parental) strains. The plaque area **(E)** and number **(F)** in the absence or presence of IAA are also depicted. The size of 150–200 plaques of each strain from 3 experiments was quantified in arbitrary units (a. u.) (*p ≤ 0.05; **p ≤ 0.01; ****p ≤ 0.0001).

To examine the relevance of *Tg*PDE1 and *Tg*PDE2, we made their conditional mutants by mAID-3HA tagging (Fig. 5A, S5A). The parasite strains were verified for localization and regulation by fluorescence imaging and western blotting. Both proteins were not detectable after incubation with IAA (Fig. 5B, S5B). Depleting *Tg*PDE1 and *Tg*PDE2 compromised the growth of mutants by ∼56 % and ∼88 % compared to the respective controls (Fig. S5C-D). A moderate but significant decline in plaque numbers was also observed (Fig. S5E), confirming a requirement of each enzyme for the optimal reproduction of tachyzoites. Nonetheless, the residual growth of the conditional strains encouraged us to make a double mutant of *Tg*PDE1 and *Tg*PDE2, as described above for *Tg*PDE8 and *Tg*PDE9 (Fig. 4), and test their physiological cooperativity (Fig. 5A). Our attempts to ablate the *Tg*PDE2 locus in the *Tg*PDE1-mAID-3HA strain were futile; however, we could delete the *Tg*PDE1 gene in the *Tg*PDE2-mAID-3HA mutant (Fig. 5C). As anticipated, in plaque assays (Fig. 5D-F), the parental strain was not affected. In contrast, the *Tg*PDE2-mAID-3HA mutant showed strongly reduced growth (∼80 %) upon IAA exposure. Equally, deletion of *Tg*PDE1 in the *Tg*PDE2-mAID-3HA strain impaired the Δ*Tg*PDE1/*Tg*PDE2-mAID-3HA mutant (−IAA sample, Fig. 5D-F). Importantly, a collective loss of both proteins aborted the parasite growth, as judged by the absence of plaques in the auxin-exposed double mutant (+IAA, Fig. 5D-F).

**Fig. 6:**
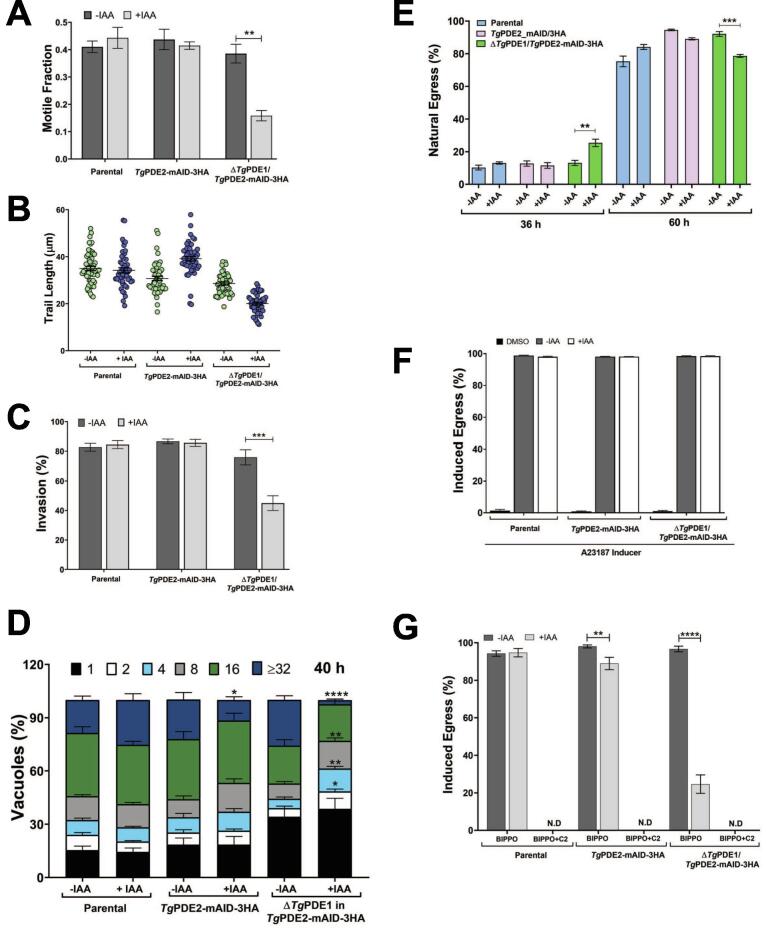
A collective loss of *Tg*PDE1 and *Tg*PDE2 disrupts the lytic cycle. **(A-B)** Gliding motility of the *Tg*PDE2-mAID-3HA and Δ*Tg*PDE1/*Tg*PDE2-mAID-3HA mutants compared to the parental (*RH*Δ*ku80*Δ*hxgprt*-TIR1) strain. The motile fraction **(**700 parasites) and trail length (150–250 trails) of each parasite strain (+/- IAA) were scored by the ImageJ program. **(C)** Invasion efficiency of the shown strains in the absence or presence of IAA. 1500–2000 parasites of each strain were imaged to compute the invasion rates. **(D)** Intracellular replication (40 h infection). Cell division was scored by counting tachyzoites within their parasitophorous vacuoles (600–700). **(E)** Natural egress from the host cells. The phenotype was measured by calculating the fraction of ruptured vacuoles (500–600) 36 h and 60 h post-infection. **(F, G)** Effect of A23187 (Ca^++^ ionophore, 2 µM), BIPPO (PDE inhibitor, 10 µM) and Compound 2 (C2, PKG inhibitor, 2 µM) on the egress of tachyzoites (MoI, 3; 24 h infection). Treatment with IAA, as applicable, was initiated 24 h before setting up the motility and invasion assays, whereas it was added 2 h post-infection during the replication and egress experiments to alleviate the effect of motility and invasion defects. Graphs display the mean ± SE (n = 5 for *A-C*, n = 6 for *D*, n = 3 for *E-G*; *p ≤ 0.05; **p ≤ 0.01; ***p ≤ 0.001; ****p ≤ 0.0001).

We next performed detailed phenotyping of the Δ*Tg*PDE1/*Tg*PDE2-mAID-3HA strain to evaluate the lytic cycle events, such as gliding motility, invasion, replication, and egress (Fig. 6). In contrast to the parental and *Tg*PDE2-depleted strains, the double mutant's motile fraction, trail length, and invasion efficiency were strongly reduced after auxin treatment (Fig. 6A-C). Notably, the deletion of *Tg*PDE1 (−IAA sample) exerted no evident defect across these features. In addition, knockdown of *Tg*PDE2 alone only moderately attenuated the parasite replication; however, the double mutant treated with auxin showed an extreme decline in the number of proliferating parasites, as scored by the size of parasitophorous vacuoles (Fig. 6D). Intriguingly, the depletion of *Tg*PDE2 in the Δ*Tg*PDE1/*Tg*PDE2-mAID-3HA strain exerted opposing effects on the parasite egress in early and late cultures (Fig. 6E), which was increased 36 h post-infection but declined after 60 h. No egress defect was observed in the *Tg*PDE2-mAID-3HA strain at any tested time points. These phenotypic assays underpin the singular and collective significance of *Tg*PDE1 and *Tg*PDE2 enzymes and highlight their mutual interplay during the lytic cycle.

### 2.6 The Δ*Tg*PDE1/*Tg*PDE2-mAID-3HA strain is refractory to BIPPO-induced egress

To further understand the role of signaling during egress, we deployed a chemogenetic approach utilizing a calcium ionophore (A23187) and inhibitors of “*cGMP-specific PDE*” (BIPPO) as well as PKG (C2 or Compound 2), all of which are widely used to understand the biology of *T. gondii*. We tested their impact on egress of the parental, *Tg*PDE2-mAID-3HA and Δ*Tg*PDE1/*Tg*PDE2-mAID-3HA strains cultured without or with auxin (Fig. 6F-G). As envisaged, A23187, an ionophore activating calcium signaling downstream of cyclic nucleotides [21], [22], [23], [24], triggered a complete egress of the three strains irrespective of the IAA treatment (Fig. 6F). Equally, BIPPO induced almost total lysis of all samples but the auxin-treated double mutant, which responded by only 20–25 % egress (Fig. 6G). In light of enzyme inhibition assays (Fig. 3A), these data entail *Tg*PDE1 as a primary target of BIPPO. The resistance of auxin-treated double mutant to this drug appeared to be a combined outcome of *Tg*PDE1 deletion (*i.e.*, the absence of drug target) and rise in cAMP after knockdown of *Tg*PDE2, leading to suboptimal activation of PKG and hyper-activation of PKA. We, therefore, examined the effect of PKG inhibitor, Compound 2 [25] on BIPPO-induced egress (Fig. 6G). The residual egress of the auxin-treated double mutant was indeed completely blocked by Compound 2, suggesting that the process is mediated by PKG.

### 2.7 Ultrastructural imaging of intracellular tachyzoites

**Fig. 7:**
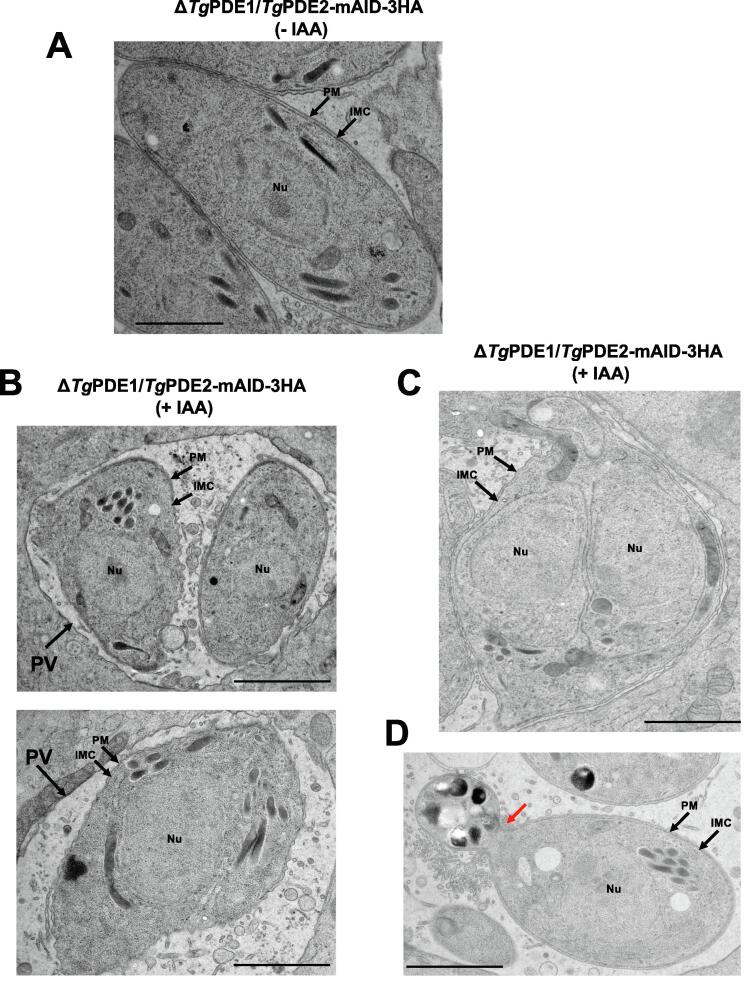
Ultrastructural imaging of the Δ*Tg*PDE1/*Tg*PDE2-mAID-3HA mutant by transmission electron microscopy. **(A)** Intracellular tachyzoites cultured without IAA (control). **(B-D)** Tachyzoites of the double mutant after treatment with IAA (24 h). Scale bar: 500 nm. *Abbreviations*: PV: parasitophorous vacuoles; IMC: inner membrane complex; PM: plasma membrane; Nu: Nucleus.

We performed the transmission electron microscopy of the Δ*Tg*PDE1/*Tg*PDE2-mAID-3HA strain to gain ultrastructural insight into the phenotype (Fig. 7). The double mutant cultured without auxin had a normal morphology with intact organelles, excluding a detrimental effect of *Tg*PDE1 deletion (Fig. 7A). In contrast, a knockdown of *Tg*PDE2 resulted in an aberrant/distorted shape of tachyzoites (Fig. 7B). Besides a much lower number of parasites per vacuole, we noted enlarged vacuolar space and impaired budding of progeny. The endodyogeny was arrested in auxin-treated cultures (Fig. 7C). We also observed a population of abnormal-shaped tachyzoites with constricted terminal regions (see red arrow in Fig. 7D). In further assays, we utilized immunogold labeling of *Tg*PDE1 and *Tg*PDE2 to decipher their spatial distribution. Given the distinct apical presence of *Tg*PDE9, we included it as a control for potential sample processing artifacts that may cause mislocalization of PDEs (Fig. S6A-C). The quantification of images disclosed that a majority of parasites (>60 %) expressed *Tg*PDE9-smHA in the conoid region at the apical pole near the plasmalemma (Fig. S6A). *Tg*PDE1-smHA was detected mainly at the cytosolic periphery (38 %) and inner membrane complex (27 %), whereas *Tg*PDE2-smHA was expressed in the cytosol (45 %), dense granules (24 %) and rhoptries (15 %).

### 2.8 *Tg*PDE1 and *Tg*PDE2 may be regulated by a specific kinase and phosphatase network

Our final assays explored the interaction network of *Tg*PDE1 and *Tg*PDE2 in *T. gondii* (Fig. S7). We precipitated PDEs and their protein binding partners using α-HA agarose beads and subjected them to liquid chromatography-mass spectrometric analysis. The parental strain was included as a negative control. The principal component analysis endorsed the proteomic dataset’s technical and biological reproducibility, as illustrated by the grouping of samples in each cohort (Fig. S7A). The heatmap also displayed evident clusters of proteins binding to *Tg*PDE1 and *Tg*PDE2 but absent in the control sample (Fig. S7B). We detected 143 and 22 unique interactors of *Tg*PDE1 and *Tg*PDE2, respectively (*p* ≤ 0.01, at least twofold enriched compared to the parental control). In total, 38 proteins were bound to both phosphodiesterases (Fig. S7C). Importantly, no other PDE except the bait was present in immunoprecipitated samples (Table S1), validating the quality of enzyme assays involving *Tg*PDE1 and *Tg*PDE2. Based on our current understanding of PDEs in other model organisms and their contextual relevance to signaling in apicomplexan parasites, we shortlisted interacting proteins, including a group of kinases and serine/threonine phosphatases (Fig. S7D). We suspect that some of the proteins identified herein as potential interaction partners may be involved in regulating *Tg*PDE1 and *Tg*PDE2 catalysis (for a complete list, see Table S1).

## 3 Discussion

*Toxoplasma gondii* has evolved an expanded panel of highly divergent phosphodiesterases to counter-regulate the cyclic nucleotide signaling. All 18 PDEs encoded by its genome belong to the class I phosphodiesterases [13], [14]. Here, we characterized the substrate specificity, physiological relevance, functional redundancy and spatial distribution of PDEs during the lytic cycle of *T. gondii*. This study reports the catalytic activity of 11 enzymes expressed in the tachyzoite stage. Except for *Tg*PDE2 hydrolyzing only cAMP, others are promiscuous dual-specific proteins degrading cAMP and cGMP. Therefore, unlike its mammalian host [26], [27] and related parasite *P. falciparum* [28], [29], *T. gondii* harbors a much larger set of dual-specific enzymes. We also found that *Tg*PDE1 exhibits a 4x higher affinity for cGMP than cAMP. The mutagenesis, phenotyping and localization datasets reveal functional cooperation of *Tg*PDE1 and *Tg*PDE2 during the lytic cycle. Some of the findings above echo a recent study [15] overlapping with this and our earlier work [14].

The K_m_ values of *Tg*PDEs range from 14 to 118 µM, comparable to most class I phosphodiesterases. For example, of the 11 human PDE families, *h*PDE3 to *h*PDE11 display K_m_ of 0.04–9 µM, whereas *h*PDE1 and hPDE2 are within 10 to 100 µM [30]. Our work also sheds light on the *modus operandi* of PDE inhibitors frequently used in parasitology research. We observed that BIPPO could potently inhibit cGMP hydrolysis by *Tg*PDE1 and *Tg*PDE7; however, it is the former enzyme that underlies the drug's effect during the lytic cycle. Moreover, another physiologically-critical enzyme, *Tg*PDE2, is refractory to inhibition by BIPPO and zaprinast. Both drugs can inhibit cAMP catalysis by *Tg*PDE9, although the dispensability of this protein excludes it as a drug target in tachyzoites. Potent inhibition of cAMP hydrolysis compared to cGMP by dual-specific *Tg*PDE1 and *Tg*PDE9 in BIPPO and zaprinast-treated samples can be explained by their differential affinity (K_m_) for these substrates. Consistent with homology modeling of the *Toxoplasma* PDEs [13], [14] and phenotypic studies performed in *Plasmodium falciparum* [29], our enzyme assays suggest that the two alleged *cGMP-specific* PDE inhibitors also perturb the cAMP pathway besides cGMP signaling. Thus, the enzyme kinetics and chemogenetic phenotyping presented herein offer a renewed prospect for developing novel PDE inhibitors and parasite-specific therapeutics.

This work uncovers remarkable plasticity in the counter-regulation of cyclic nucleotide signaling, as exemplified by catalysis and mutagenesis of *Tg*PDE1-2 and *Tg*PDE7-9 proteins. Tachyzoites can survive the absence of *Tg*PDE7, *Tg*PDE8 and *Tg*PDE9 enzymes, whereas they depend on the cooperation of *Tg*PDE1 and *Tg*PDE2 for specific events during their asexual growth. Notably, neither the knockout of *Tg*PDE1 nor the knockdown of *Tg*PDE2 affects the motility and invasion, but a loss of both compromises these features. Surprisingly, the Δ*Tg*PDE1/*Tg*PDE2-mAID-3HA mutant showed a higher (premature) egress at 36 h, which reversed into a moderate impairment at 60 h, implying programmed crosstalk of cAMP and cGMP signaling as the parasite nears the end of its lytic cycle. Regarding parasite replication, mutagenesis of *Tg*PDE1 and *Tg*PDE2 exerted a negligible effect, though the simultaneous loss of both phosphodiesterases delivered a potent phenotype. Unlike *Tg*PDE1, we could not knockout *Tg*PDE2, suggesting that any other orthologs cannot fully compensate for the physiological role of the latter enzyme. A dominant expression of *Tg*PDE1 and *Tg*PDE2 in the parasite cytosol may account for their partial functional redundancy. Varied subcellular localization of PDEs in tachyzoites also reflects a compartmentalized control of cyclic nucleotide signaling, warranting further studies. Last but not least, we demonstrate *Tg*PDE1 and *Tg*PDE2 as potential drug targets to control the acute infection of *T. gondii*.

## 4 Materials and methods

### 4.1 Biological reagents

The *RH*Δ*ku80*Δ*hxgprt* [31] and *RH*Δ*ku80*Δ*hxgprt*-TIR1 [4] strains of *T. gondii* were offered by Vern Carruthers (University of Michigan, MI) and David Sibley (Washington University, St. Louis, MO), respectively. The *pU6-Universal* and *pSag1-Cas9-U6-sgUPRT* vectors for expression of Cas9 and single guide RNA (*sg*RNA), and the *pTUB1-YFP-mAID-3HA-HXGPRT* plasmid, were provided by David Sibley (Washington University, St. Louis, USA). The antibodies recognizing *Tg*Gap45 [32] and *Tg*Hsp90 [33] were donated by Dominique Soldati-Favre (University of Geneva, Switzerland) and Sergio Angel (IIB-INTECH, Buenos Aires, Argentina), respectively. Other antibodies against the HA epitope and *Tg*Sag1 were purchased from Takara-Bio (Japan) and Sigma-Aldrich (Germany). The secondary antibodies (Alexa488, Alexa594; IRDye 680RD, 800CW) and oligonucleotides (Table S2) were obtained from Thermo Fisher Scientific (Germany). The anti-HA mAb-conjugated agarose beads (clone HA-7) were procured from Sigma-Aldrich (Germany). The cell culture reagents were purchased from PAN Biotech (Germany), and other standard chemicals were supplied by Sigma-Aldrich and Carl Roth (Germany). The kits for isolation, cloning and purification of nucleic acids were acquired from Analytik Jena and Life Technologies (Germany). The PDE assay kits (colorimetric) were purchased from Abcam (UK) and Enzo Life Science (USA).

### 4.2 Host cell and parasite cultures

The human foreskin fibroblasts (HFFs; Cell Lines Service, Eppelheim, Germany) were grown to confluence and harvested for further passaging by trypsin-EDTA treatment. Cells were cultured in Dulbecco’s modified Eagle medium containing glucose (4.5 g/L), 10 % heat-inactivated fetal bovine serum (FBS; PAN Biotech), 2 mM glutamine, 1 mM sodium pyruvate, 1x minimum Eagle’s medium nonessential amino acids, penicillin (100 U/mL), and streptomycin (100 μg/mL) in a humidified incubator (37 °C, 5 % CO_2_). The tachyzoite stage of *T. gondii* was maintained by serial culture in confluent HFF monolayers using a multiplicity of infection (MoI) of 3. Parasites for all experiments were prepared by squirting infected cultures through a 27G syringe (2x) unless stated otherwise.

### 4.3 Making of transgenic parasites

To generate the *Tg*PDEx-mAID-3HA mutants (x = 1, 2 or 9), we designed *pU6-Cas9-TgPDEx*_*sgRNA*_ constructs expressing Cas9 and *sg*RNA targeting the 3′UTR of respective genes using Q5 site-directed mutagenesis kit (New England Biolabs). The CRISPR-Cas9 constructs were transfected with corresponding donor amplicons in the *RH*Δ*ku80*Δ*hxgprt*-TIR1 strain. The amplicons harbored mAID-3HA and HXGPRT selection cassette flanked by short (50 bp) 5′ and 3′ homology arms for crossovers at the respective PDE locus. Tachyzoites expressing mAID-3HA fused to the target PDE protein as well as HXGPRT were selected using mycophenolic acid (25 μg/mL) and xanthine (50 μg/mL)[34], cloned by limiting dilution. The mutant clones were screened by genomic PCR using recombination-specific primers (Table S2).

To generate the direct knockout of *Tg*PDE1 and *Tg*PDE8 in the conditional mutants of *Tg*PDE2-mAID-3HA and *Tg*PDE9-mAID-3HA, respectively, a CRISPR-Cas9 vector expressing two *sg*RNA to target the 5′UTR and 3́UTR regions of each gene was constructed. In the first step, we made two separate CRISPR-Cas9 vectors for *Tg*PDE1 and *Tg*PDE8 knockout by replacing the UPRT *sg*RNA with gene-specific *sg*RNAs targeting 5′ or 3′UTR in the *pSAG1-Cas9-U6-sgUPRT* plasmid. We then amplified the *pU6-sg3′UTR-TgPDE1/8* region (∼678 bp) using *sg*RNA2-*Kpn*I-F and *sg*RNA2-*Xho*I-R primers (Table S2) and *pSAG1-Cas9-U6-sg3́UTR-TgPDE1/8* as templates. The amplicons were ligated into *Kpn*I-*Xho*I-digested *pSAG1-Cas9-U6-sg5′UTR-TgPDE1* and *pSAG1-Cas9-U6-sg5′UTR-TgPDE8* vectors, as applicable. The subsequent dual CRISPR-Cas9 plasmids were co-transfected with respective donor amplicons. The latter comprised a DHFR-TS selection cassette flanked by 5′- and 3′-homology arms (∼70 bp) targeting the upstream and downstream *TgPDE1* and *TgPDE8* loci. Transgenic parasites were cloned using 1 μM pyrimethamine [35] and screened by genomic PCR (primers in Table S2).

### 4.4 Indirect immunofluorescence assays

As described elsewhere [36], HFF cultured on glass coverslips were infected with tachyzoites for the indicated periods. Samples were fixed with 4 % paraformaldehyde (15 min) and neutralized by 0.1 M glycine-PBS (5 min). Afterward, cells were permeabilized in 0.2 % Triton X-100/PBS (20 min), followed by blocking with 2 % bovine serum albumin (0.2 % Triton X-100/PBS) for 20 min. Samples were stained with primary antibodies (α-HA, mouse, 1:3000; α*Tg*Gap45, rabbit, 1:3000; α-*Tg*Sag1, mouse, 1:1000) for 1 h. They were washed 3x with 0.2 % Triton X-100/PBS and stained with Alexa488/594-conjugated antibodies for 45 min. After additional PBS washing, samples were finally mounted in Fluoromount G containing DAPI (Southern Biotech, Birmingham, AL) and stored in the dark at 4 °C. Images were acquired by fluorescence microscopy (Zeiss, Germany).

### 4.5 Immunoblot analysis

Tachyzoites were harvested and pelleted (800 *g*, 4 °C, 10 min), followed by washing with ice-cold PBS and re-pelleting in a 1.5 mL tube (8000 *rpm*, 3 min, 4 °C). Cells were lysed in 55 μL buffer (10 mM K_2_HPO_4_, 150 mM NaCl, 5 mM EDTA, 5 mM EGTA, pH 7.4; 0.2 % sodium deoxycholate, 1 % Triton X-100) and protease inhibitors (trypsin inhibitor, 20 µg/mL; aprotinin, 10 µg/mL; benzamidine, 500 µg/mL; PMSF, 0.5 mM; Na_3_VO_4_, 0.1 mM; NaF, 50 mM). Samples were incubated on ice for 30 min and then centrifuged (20000 *g*, 15 min, 4 °C) to collect the cell-free extract (50 μL), which were mixed with 5x loading buffer (13 μL, no boiling), followed by SDS-PAGE (6–8 %). Proteins were blotted onto a nitrocellulose membrane (85 mA/cm^2^, 2 h, semi-dry) and stained overnight at 4 °C with the mouse α-HA (1:10000) and rabbit α-*Tg*Hsp90 (1:10000) antibodies diluted in 5 % skimmed milk with 0.2 % Tween 20/TBS. Immunoblot was washed 3x with 0.2 % Tween 20/TBS (5 min) and incubated with IRDye-conjugated secondary antibodies (680RD and 800CW with 1:15000 dilution, 1 h). The antibody-stained protein bands were visualized by an Odyssey Fc imaging system (LI-COR Biosciences).

### 4.6 Lytic cycle assay

The impact of genetic manipulation on the lytic cycle of tachyzoites was determined by standard phenotyping methods, as described in our previous works [37], [38]. For plaque assay, the confluent HFF monolayers in 6-well plates were infected with 200 parasites/well and incubated for 7 to 8 days without perturbation. Samples were fixed with ice-cold methanol for 10 min and then stained with crystal violet solution for 15 min. Plaques were imaged and scored for size and number using the ImageJ software (NIH, Bethesda). To quantify the invasion efficiency, the HFF monolayers on coverslips placed in 24-well plates were infected with tachyzoites (MoI: 10) for 30 min at 37 °C, followed by fixation with 4 % paraformaldehyde/PBS and neutralization with 0.1 % glycine/PBS. Before permeabilization, samples were stained with the mouse α-*Tg*Sag1 antibody (1:1000) to visualize the non-invaded/extracellular parasites. Cells were washed 3x with PBS, permeabilized with 0.2 % Triton X-100/PBS for 20 min, and stained with the rabbit α-*Tg*Gap45 antibody (1:10000) to score the invaded parasites. The fractions of invaded/intracellular parasites were determined to compare the invasion rates across the parasite strains.

To gauge the intracellular replication of tachyzoites, HFF cells grown on coverslips were infected (MoI:1, 40 h). Samples were subjected to permeabilization, neutralization, blocking, and staining with the rabbit α-*Tg*Gap45 antibody. The tachyzoite proliferation was assessed by enumerating parasitophorous vacuoles harboring a variable number of progeny. For the egress assay, the host cells were infected (MoI:1) for 36 h and 60 h, followed by immunostaining, as done for the invasion assay. The disrupted vacuoles with egressing parasites were quantified by α-*Tg*Sag1/Alexa488 staining (green), and the fraction of intact vacuoles was scored based on α-*Tg*Gap45/Alexa594 labeling (red). To evaluate the gliding motility, parasites (4 × 10^5^) suspended in Hank’s balanced salt solution were incubated to let them settle and glide (30 min, 37 °C) on glass coverslips pre-coated with 0.01 % BSA (2 h). As mentioned elsewhere, samples were stained with α-*Tg*Sag1 and Alexa488 antibodies to visualize the gliding trails and parasites. The motile fraction was counted on the microscope, and trail lengths were quantified by the ImageJ program.

### 4.7 Immunoprecipitation of PDE proteins

The cell-free extract was prepared as described above*.* To precipitate the native PDEs, 50 µL of anti-HA agarose beads were added to the tachyzoite extract (2 mg protein). The volume was adjusted to 1 mL by a lysis buffer containing protease inhibitors. The pull-down reaction was set with constant rotation (4 °C, 4 h). Afterward, protein-conjugated beads were pelleted (200 *g*, 30 s), washed 2x with ice-cold lysis buffer with protease inhibitors, and then once with the PDE dilution buffer (150 mM NaCl and 10 mM Tris-HCl, pH 7.4) [29]. Samples were given a final wash with 10 mM Tris-HCl buffer (pH 7.4) before using them for the enzyme assays.

### 4.8 PDE enzyme assay

The experiment was performed using colorimetric kits (Enzo Life Science, Netherlands; Abcam, UK) based on the enzymatic cleavage of 3′5′cAMP/3′5′cGMP to 5′AMP/5′GMP, which are further hydrolyzed by 5′-nucleotidase to their nucleoside and phosphate moieties. The phosphate group is quantified to determine the PDE activity. To set up the assay, immunoprecipitated proteins (1–10 µg) were suspended in the reaction buffer (50 μL), followed by the addition of cAMP or cGMP (200 μM). Samples were incubated at 37 °C for 1 h and mixed with 100 μL of the green reagent (30 min, room temperature) to terminate the reaction. Subsequently, the OD_620_ was measured to quantify the phosphate group. We also included a cAMP-specific PDE from the bovine brain as a positive control and several negative controls (no protein, no substrate) for validation purposes. The standards with varying phosphate amounts (0.25–4 nmol) provided by the kit were included in all experiments to quantify the enzymatic hydrolysis of cAMP and cGMP.

To determine the kinetic parameters (K_m_, V_max_, IC_50_) of selected phosphodiesterases (*Tg*PDE1, *Tg*PDE2, *Tg*PDE7, *Tg*PDE9), assays were standardized for the protein amount and incubation period. The substrate dependence was tested in the linear range of reaction time and amount of each PDE. The K_m_ and V_max_ were calculated by the Michaelis-Menten equation using the GraphPad Prism suite. We also attempted to examine the kinetics of *Tg*PDE8 (dual-specific, apical location); however, its low expression and poor catalytic activity prevented us from determining reproducible K_m_ values. Our additional assays tested the PDE inhibition by 3-isobutyl-1-methylxanthine (IBMX, 100 µM), 1,4-Dihydro-5-(2-propoxyphenyl)-7H-1,2,3- triazolo (4,5-d) pyrimidin-7-one (zaprinast, 300 µM), 5-benzyl-3-isopropyl-1H-pyrazolo (4,3-d) pyrimidin-7(6H)-one (BIPPO, 100 µM) [13] and PF-04957325 (50 µM, Pfizer Inc). For the IC_50_ estimation, different concentrations of BIPPO (1–100 µM) and zaprinast (1–350 µM) were used. The key reaction parameters (protein amount, time, substrate) were optimized prior to the inhibition kinetics of phosphodiesterases.

### 4.9 Proteolytic digestion for mass spectrometry

As described elsewhere [39], [40], samples were processed by a single-pot solid-phase-enhanced preparation method. In brief, anti-HA agarose beads were incubated for 15 min at 60 °C in an SDS-containing buffer (1 % w/v SDS, 50 mM HEPES, pH 8.0) to release proteins, which were afterward reduced and alkylated by dithiothreitol and iodoacetamide, respectively. They were supplemented with 2 µL of carboxylate-modified paramagnetic beads (Sera-Mag SpeedBeads, GE Healthcare, 0.5 μg solids/μL water), followed by adding acetonitrile to a final concentration of 70 % (v/v). Beads were allowed to settle for 20 min at room temperature. Subsequently, samples were washed twice with 70 % (v/v) ethanol in water and once with acetonitrile. Beads were suspended in 50 mM NH_4_HCO_3_ supplemented with trypsin (Mass Spectrometry Grade, Promega) at an enzyme-to-protein ratio of 1:25 (w/w) and incubated overnight at 37 °C. Acetonitrile was added to the samples to reach a final concentration of 95 % (v/v), followed by incubation at room temperature for 20 min. To maximize the yield, supernatants derived from this initial peptide-binding step were subjected to the peptide purification procedure [40]. Each sample was washed with acetonitrile. Paramagnetic beads from the original reaction and corresponding supernatants were pooled in 2 % (v/v) dimethyl sulfoxide in water and sonicated for 1 min. After centrifugation (12500 *rpm*, 4 °C), supernatants containing tryptic peptides were transferred into a glass vial for mass spectrometry analysis and acidified with 0.1 % (v/v) formic acid.

### 4.10 Liquid chromatography-mass spectrometry analysis

Tryptic peptides were separated using an Ultimate 3000 RSLCnano LC system (Thermo Fisher Scientific) equipped with a PEPMAP100, C18, 5 µm, 0.3 × 5 mm trap (Thermo Fisher Scientific) and an HSS-T3 C18, 1.8 μm, 75 μm × 250 mm analytical reversed-phase column (Waters Corporation). Mobile phase A was water containing 0.1 % (v/v) formic acid and 3 % (v/v) DMSO. Peptides were separated using a gradient of 2–35 % mobile phase B (0.1 % v/v formic acid, 3 % v/v DMSO in acetonitrile) over 40 min at a flow rate of 300 nL/min. The total analysis time was 60 min including the wash and column re-equilibration (temperature, 55 °C). Mass spectrometric analysis of eluting peptides was conducted on an Orbitrap Exploris 480 instrument platform (Thermo Fisher Scientific). The spray voltage was set to 1.8 kV, the funnel RF level to 40, and the capillary temperature was at 275 °C. Data were acquired in data-dependent acquisition mode targeting the 10 most abundant peptides for fragmentation (Top10). Full MS resolution was set to 120,000 at *m*/*z* 200, and full MS automated gain control (AGC) target to 300 % with a maximum injection time of 50 ms. The mass range was adjusted to *m*/*z* 350–1500. For MS2 scans, the collection of isolated peptide precursors was limited by an ion target of 1x10^5^ (AGC target value of 100 %) and maximum injection times of 25 ms. The fragment ion spectra were acquired at a resolution of 15,000 at *m*/*z* 200, and the intensity threshold was kept at 1E4. The isolation window width of the quadrupole was set to 1.6 *m*/*z,* and the normalized collision energy was fixed at 30 %. All data were acquired in profile mode using positive polarity.

### 4.11 Data analysis and label-free quantification

The raw data acquired with the Exploris 480 were processed by MaxQuant (v2.0.1) suite [41], [42] using standard settings and label-free quantification (LFQ) enabled for each parameter group, *i.e.*, control and affinity-purified samples (LFQ min ratio count 2, stabilize large LFQ ratios disabled, match-between-runs). Data were searched against *T. gondii* proteome (UniprotKB/TrEMB, 8450 entries, UP000005641) and common contaminants. For peptide identification, trypsin was set as a protease, allowing for two missed cleavages. Carbamidomethylation was programmed as fixed, and methionine oxidation and acetylation of protein *N*-termini were set as variable modifications. Only peptides with a length of 7 amino acids or more were considered. Peptide and protein false discovery rates (FDR) were 1 %. In addition, proteins were identified by the presence of at least two peptides. Statistical analysis was conducted using the student’s *t*-test, which was corrected by the Benjamini–Hochberg method for multiple hypothesis testing (FDR, 0.01). Proteins with a minimum twofold enrichment in the affinity-enriched samples were considered.

### 4.12 Transmission electron microscopy

#### 4.12.1 Immunogold labeling

The immunogold analysis was carried out according to Tokuyasu [43], [44]. Confluent HFF monolayers infected by parasites (MoI:4, 24 h) were fixed with 2 % paraformaldehyde/0.1 % glutaraldehyde in 100 mM sodium phosphate buffer (pH 7.4) for 2 h at room temperature. Samples were scraped and pelleted by centrifugation (6000 *g*, 1.5 min). The cell pellets were infiltrated gradually in gelatin (1 %, 5 %, 10 % gelatin in 100 mM PB buffer, pH 7.4 at 37 °C). Subsequently, samples were cooled down on the ice to solidify gelatin and cut into small pieces, which were infiltrated overnight at 4 °C in 100 mM PB buffer containing 2.3 M sucrose (pH 7.4). Sample blocks (∼700 µm^3^) were mounted on aluminum pins and placed into the cryo-chamber, precooled to −110 °C of a cryo-ultramicrotome (UC7, Leica Microsystems, Wetzlar). Ribbons of 60 nm thin sections were picked by a Perfect Loop® with 1 % (w/v) methylcellulose and 2.3 M sucrose (1:1) in 100 mM PB buffer (pH 7.4).

For immunolabeling, grids were rinsed with a series of droplets (0.1 % glycine in PBS, pH 7.4), followed by blocking with 1 % BSA in PBS (pH 7.4). Samples were incubated for 60 min with the primary antibody (α-HA mouse, Biolegend 90150), diluted 1:100 in 1 % BSA, 0.2 % fish skin gelatin in PBS (pH 7.4). After washing with 0.1 % BSA in PBS (pH 7.4), they were incubated for 30 min with rabbit anti-mouse bridge antibody (BioZol original from Jackson-Immuno, 315–005-048), diluted 1:100 in 1 % BSA, 0.2 % fish skin gelatin in PBS (pH 7.4). Following washing, grids were treated for 20 min with Protein-10 nm A gold (CMC-Utrecht, batch 08–2021), diluted 1:25 in 1 % BSA in PBS (pH 7.4). Samples were washed (PBS and water) and then stained in 2 % uranyl oxalate (pH 7.0), followed by 10 min incubation in 1.8 % (v/w) methylcellulose/0.4 % uranyl acetate (pH 4.0, mixed 1 + 1 on ice in the dark). Grids were looped out and dried in the residual thin film of 1.8 % methylcellulose/0.4 % uranyl acetate (pH 4.0). Sections were imaged by electron microscopes JEM 2100Plus at 200 kV (JEOL, Japan) equipped with a XAROSA CMOS 20 Megapixel Camera (Emsis GmbH, Germany) or at 80 kV by Zeiss TEM 902 (Germany). Quantification of gold labeling in different organelles of tachyzoites was performed by analyzing 157 (*Tg*PDE1), 108 (*Tg*PDE2) and 62 (*Tg*PDE9) images.

#### 4.12.2 High-pressure freezing and freeze substitution

HFFs cultured on sapphire disks (3 mm, coated with 0.01 % poly-l-lysine) were infected (MoI:4). For high-pressure freezing (HPF), sapphire disks were dipped into 1-hexadecene and placed onto a flat aluminum planchette (3 mm diameter) with cells facing upwards, which was then covered with another aluminum planchette (3 mm diameter, cavity 40 µm). The planchette sandwich was placed in an HPF holder and frozen using a Wohlwend HPF Compact 03 high-pressure freezer (Engineering Office M. Wohlwend GmbH, Switzerland). The frozen samples were stored in liquid nitrogen until freeze substitution (FS). For FS, the aluminum planchettes were opened in liquid nitrogen and separated from the sapphire disks, which were then immersed in a substitution solution containing 1 % osmium tetroxide, 0.1 % uranyl acetate and 3 % H_2_O in anhydrous acetone pre-cooled to −90 °C. The FS was performed in a Leica EM AFS2 (Germany) following the protocol of 30 h (-90 °C), 12 h (-60 °C), 12 h (-30 °C) and 1 h (0 °C). Samples were washed 5x with anhydrous acetone, stepwise embedded in EPON 812 mixed with acetone (30 %, 60 %, 100 %) and finally polymerized for 48 h at 60 °C. Ultrathin sections of 70 nm were prepared using a Leica UC7 ultramicrotome (Germany) and a 35° Ultra diamond knife (DiATOME, Switzerland). Sections were collected on formvar-coated grids and stained for 30 min with 2 % uranyl acetate and 20 min with 3 % lead citrate (Roth, Germany). Images were collected using the JEM 2100Plus system (200 kV, JEOL, Japan), equipped with a XAROSA CMOS 20MP camera (Emsis, Germany).

## 5 Data analysis, availability, and presentation

All assays were executed at least three independent times unless specified otherwise. The mass spectrometry data were processed using proprietary programs associated with each instrument. The datasets have been deposited to the ProteomeXchange Consortium (PXD032173) *via* the jPOST partner repository (JPST001521) (http://proteomecentral.proteomexchange.org, https://doi.org/10.1093/nar/gkw1080). Other results presented herein were analyzed and plotted using the GraphPad Prism v8 software. The error bars in graphs signify means with SE. The *p*-values were computed by Student's *t*-test (*p ≤ 0.05; **p ≤ 0.01; ***p ≤ 0.001; ****p ≤ 0.0001). Images of transgenic strains and phenotyping assays (plaque, immunofluorescence, immunoblot, PCR *etc.*) show only a representative of the three or more biological replicates.

## Declaration of Competing Interest

The authors declare that they have no known competing financial interests or personal relationships that could have appeared to influence the work reported in this article.
