# Supplementary material for: Plasticity and therapeutic potential of cAMP and cGMP-specific phosphodiesterases in Toxoplasma gondii
Source: Comput Struct Biotechnol J. 2022 Sep 24;20:5775–89. doi: 10.1016/j.csbj.2022.09.022 (PMC9619220; doi:10.1016/j.csbj.2022.09.022)
Supplement: Supplementary data 3 [file mmc3.pdf]

Figure S1

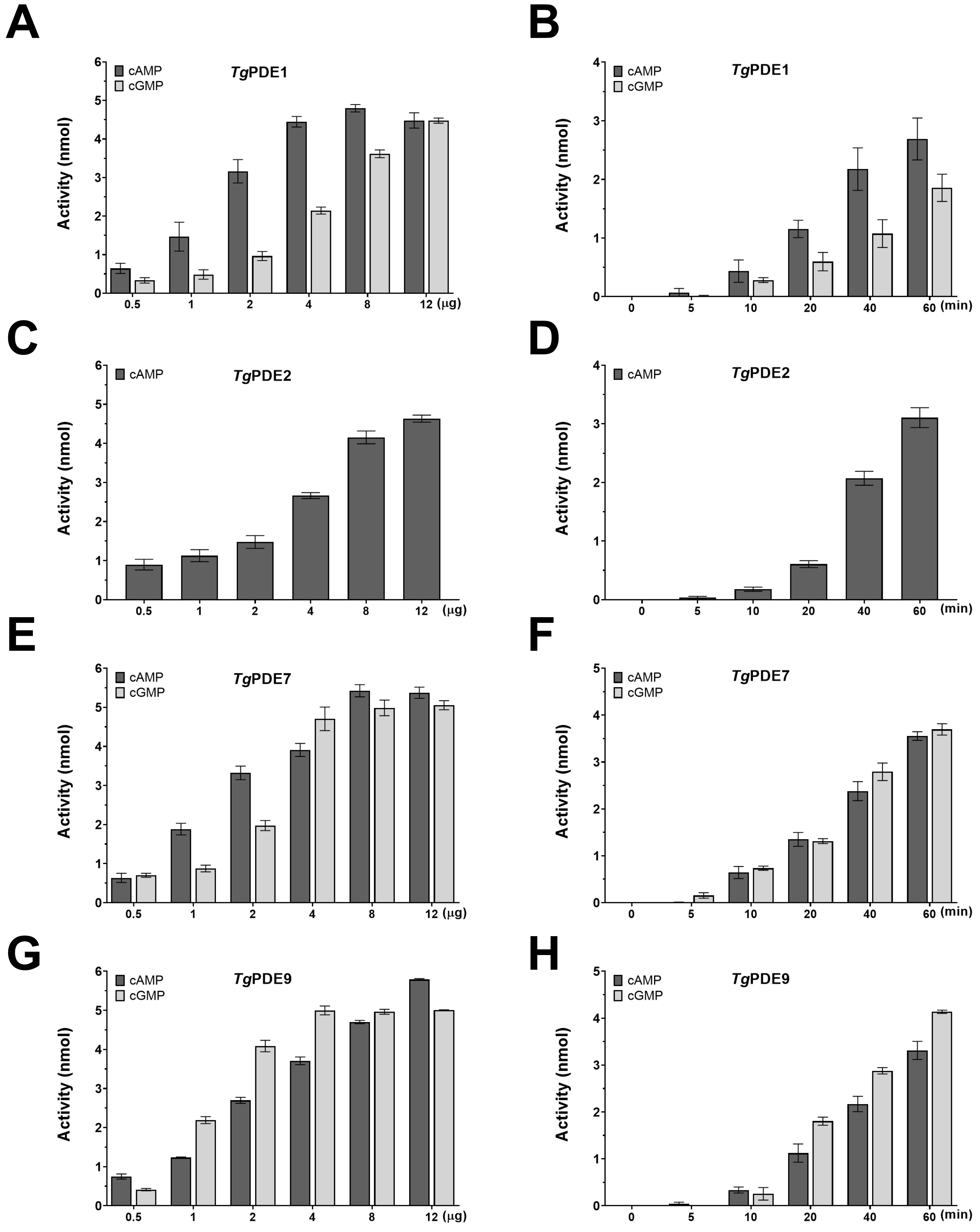
A
B
C
D
E
F
G
H
TgPDE1
TgPDE2
TgPDE7
TgPDE9
cAMP
cGMP
Activity (nmol)
0.5
1
2
4
8
12 (μg)
0
5
10
20
40
60 (min)

Figure S2

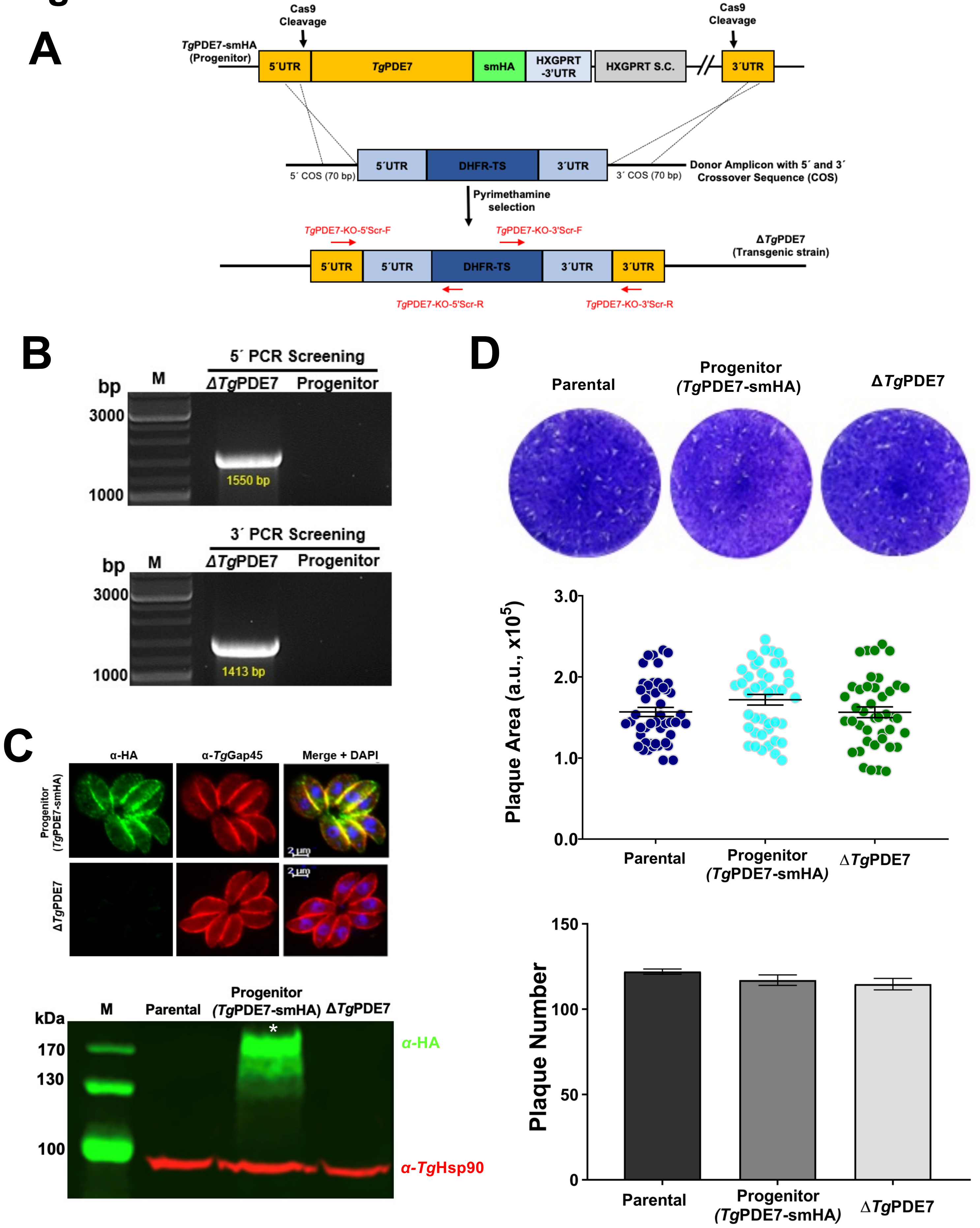
A
Cas9 Cleavage
TgPDE7-smHA (Progenitor)
5´UTR
TgPDE7
smHA
HXGPRT -3'UTR
HXGPRT S.C.
3´UTR
5´COS (70 bp)
DHFR-TS
3´COS (70 bp)
Donor Amplicon with 5´ and 3´ Crossover Sequence (COS)
Pyrimethamine selection
TgPDE7-KO-5'Scr-F
TgPDE7-KO-3'Scr-F
TgPDE7-KO-5'Scr-R
TgPDE7-KO-3'Scr-R
ΔTgPDE7 (Transgenic strain)
B
5´ PCR Screening
bp
M
ΔTgPDE7
Progenitor
3000
1000
1550 bp
3´ PCR Screening
1413 bp
C
α-HA
α-TgGap45
Merge + DAPI
Progenitor (TgPDE7-smHA)
ΔTgPDE7
2 µm
kDa
Parental
Progenitor (TgPDE7-smHA)
170
130
100
α-HA
α-TgHsp90
D
Parental
Progenitor (TgPDE7-smHA)
ΔTgPDE7
Plaque Area (a.u., x10^5)
3.0
2.0
1.0
0.0
Plaque Number
150
100
50
0

Figure S3

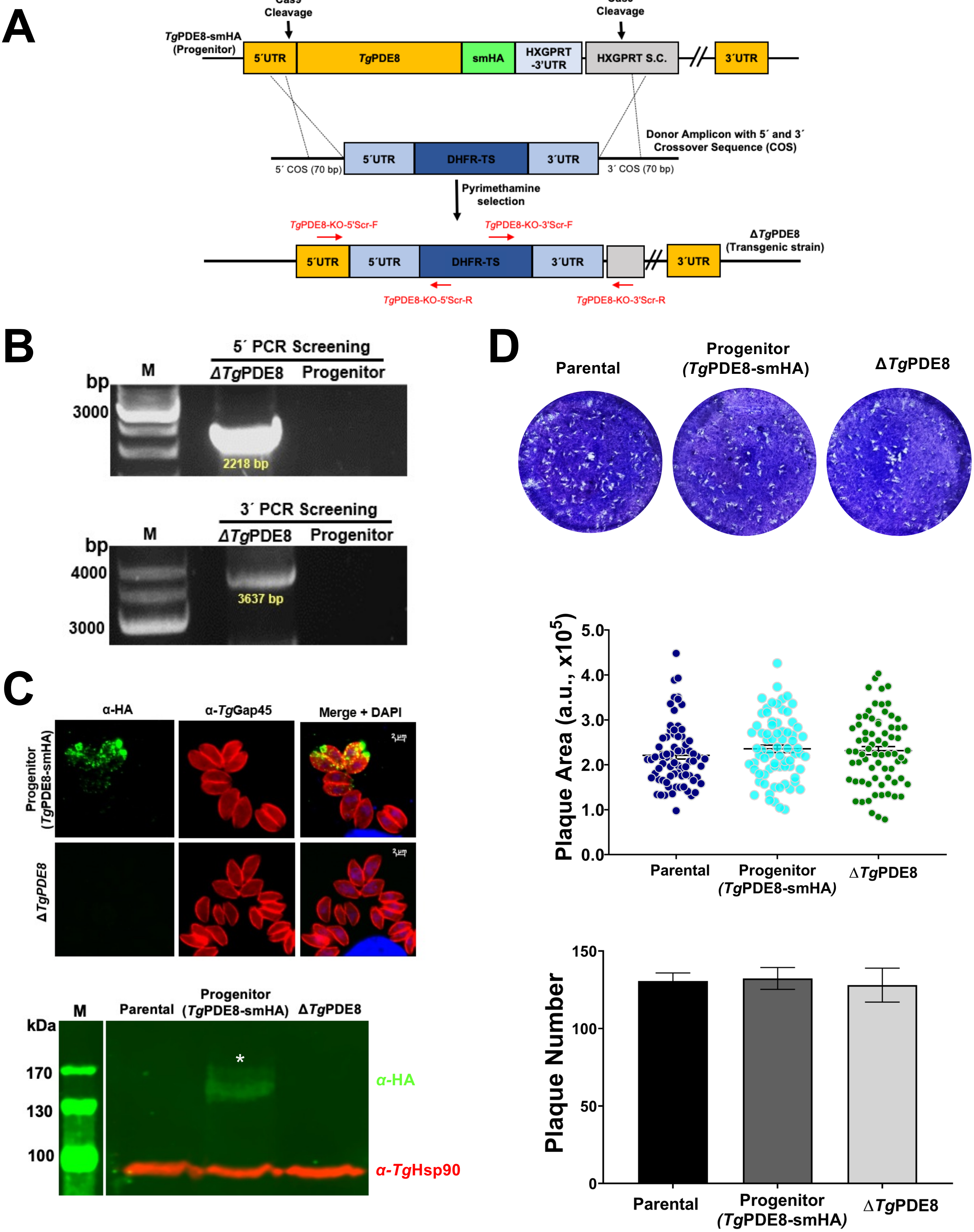

A
Cas9 Cleavage
TgPDE8-smHA (Progenitor)
5´UTR
TgPDE8
smHA
HXGPRT -3'UTR
HXGPRT S.C.
3´UTR
Donor Amplicon with 5´ and 3´ Crossover Sequence (COS)
5´ COS (70 bp)
DHFR-TS
3´ COS (70 bp)
Pyrimethamine selection
TgPDE8-KO-5'Scr-F
TgPDE8-KO-3'Scr-F
ΔTgPDE8 (Transgenic strain)
TgPDE8-KO-5'Scr-R
TgPDE8-KO-3'Scr-R
B
5´ PCR Screening
M
ΔTgPDE8
Progenitor
bp
3000
2218 bp
3´ PCR Screening
4000
3637 bp
3000
C
α-HA
α-TgGap45
Merge + DAPI
Progenitor (TgPDE8-smHA)
ΔTgPDE8
2μm
kDa
Parental
Progenitor (TgPDE8-smHA)
170
130
100
*
α-HA
α-TgHsp90
D
Parental
Progenitor (TgPDE8-smHA)
ΔTgPDE8
Plaque Area (a.u., x10^5)
5.0
4.0
3.0
2.0
1.0
0.0
Plaque Number
150
100
50
0

Figure S4

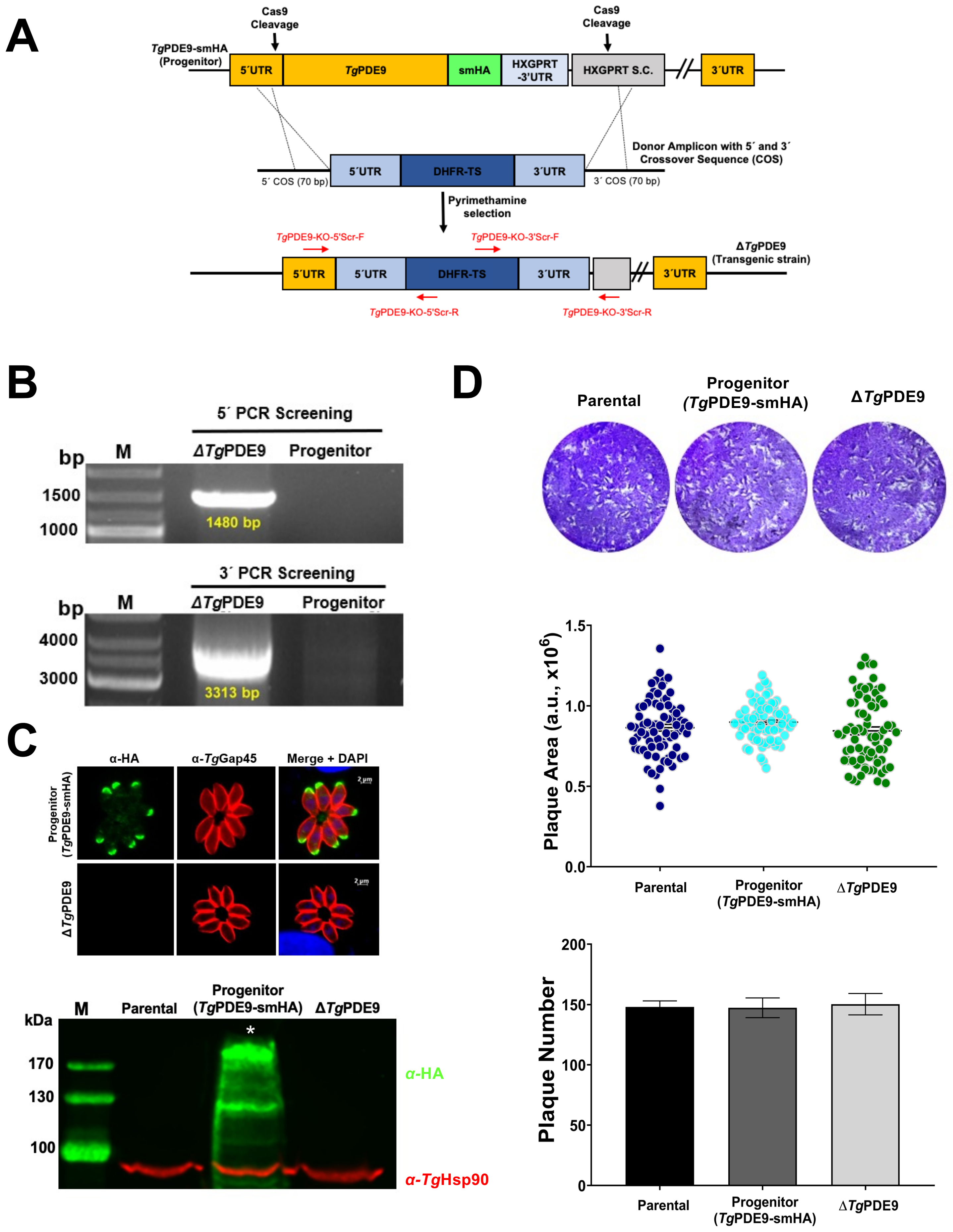
A
Cas9 Cleavage
Cas9 Cleavage
TgPDE9-smHA (Progenitor)
5´UTR
TgPDE9
smHA
HXGPRT -3'UTR
HXGPRT S.C.
3´UTR
Donor Amplicon with 5´ and 3´ Crossover Sequence (COS)
5´UTR
DHFR-TS
3´UTR
5´ COS (70 bp)
3´ COS (70 bp)
Pyrimethamine selection
TgPDE9-KO-5'Scr-F
TgPDE9-KO-3'Scr-F
ΔTgPDE9 (Transgenic strain)
TgPDE9-KO-5'Scr-R
TgPDE9-KO-3'Scr-R
B
5´ PCR Screening
bp
M
ΔTgPDE9
Progenitor
1500
1000
1480 bp
3´ PCR Screening
4000
3000
3313 bp
C
α-HA
α-TgGap45
Merge + DAPI
Progenitor (TgPDE9-smHA)
ΔTgPDE9
2 µm
kDa
Parental
Progenitor (TgPDE9-smHA)
170
130
100
*
α-HA
α-TgHsp90
D
Parental
Progenitor (TgPDE9-smHA)
ΔTgPDE9
Plaque Area (a.u., x10⁶)
1.5
1.0
0.5
0.0
Plaque Number
200
150
100
50
0

Figure S5

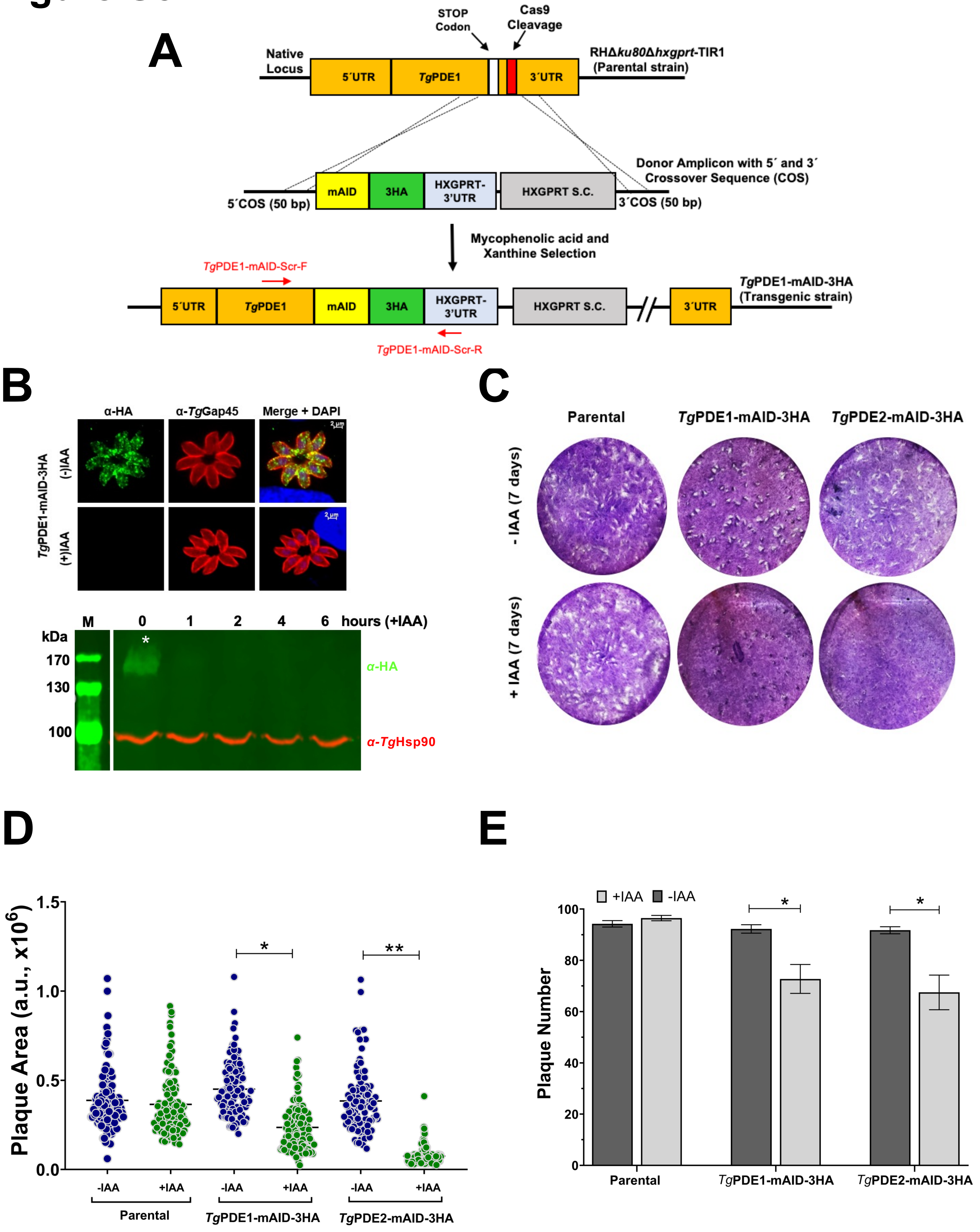
A
STOP Codon
Cas9 Cleavage
Native Locus
5´UTR
TgPDE1
3´UTR
RHΔku80Δhxgprt-TIR1 (Parental strain)
Donor Amplicon with 5´ and 3´ Crossover Sequence (COS)
mAID
3HA
HXGPRT-3'UTR
HXGPRT S.C.
5´COS (50 bp)
3´COS (50 bp)
Mycophenolic acid and Xanthine Selection
TgPDE1-mAID-Scr-F
TgPDE1-mAID-Scr-R
TgPDE1-mAID-3HA (Transgenic strain)
B
α-HA
α-TgGap45
Merge + DAPI
TgPDE1-mAID-3HA
(-)IAA
(+)IAA
M
0
1
2
4
6
hours (+IAA)
kDa
170
130
100
α-HA
α-TgHsp90
C
Parental
TgPDE1-mAID-3HA
TgPDE2-mAID-3HA
- IAA (7 days)
+ IAA (7 days)
D
Plaque Area (a.u., x10^6)
1.5
1.0
0.5
0.0
-IAA
+IAA
Parental
TgPDE1-mAID-3HA
TgPDE2-mAID-3HA
E
+IAA
-IAA
Plaque Number
100
80
60
40
20
0
Parental
TgPDE1-mAID-3HA
TgPDE2-mAID-3HA

Figure S6

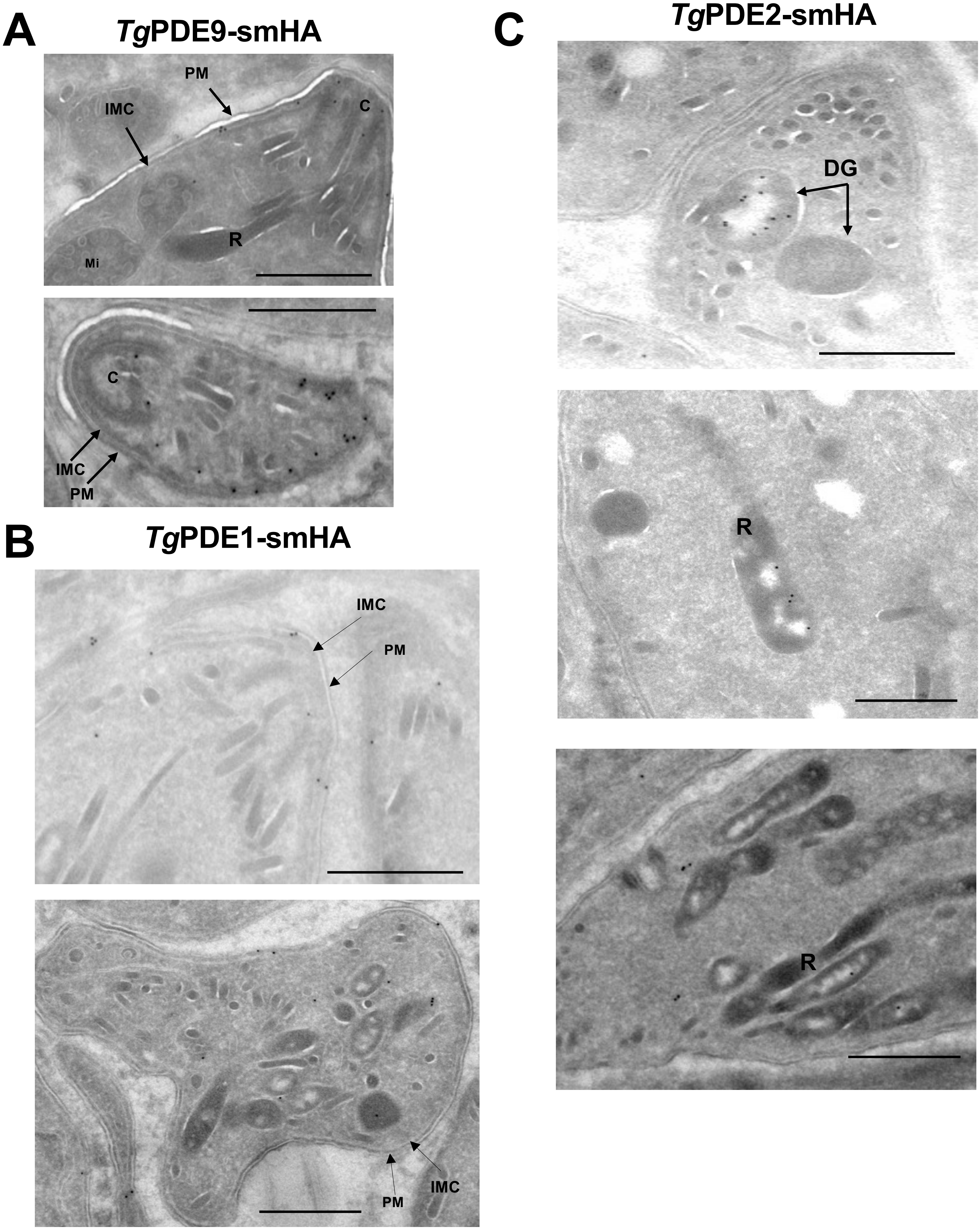
A
TgPDE9-smHA
PM
IMC
C
R
Mi
C
IMC
PM
B
TgPDE1-smHA
IMC
PM
PM
IMC
C
TgPDE2-smHA
DG
R
R

Figure S7

A

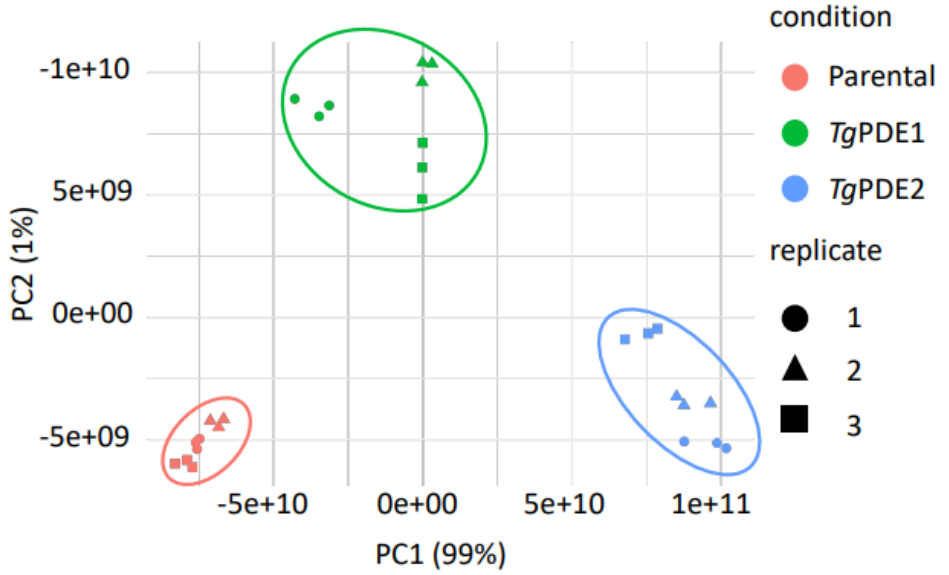

B

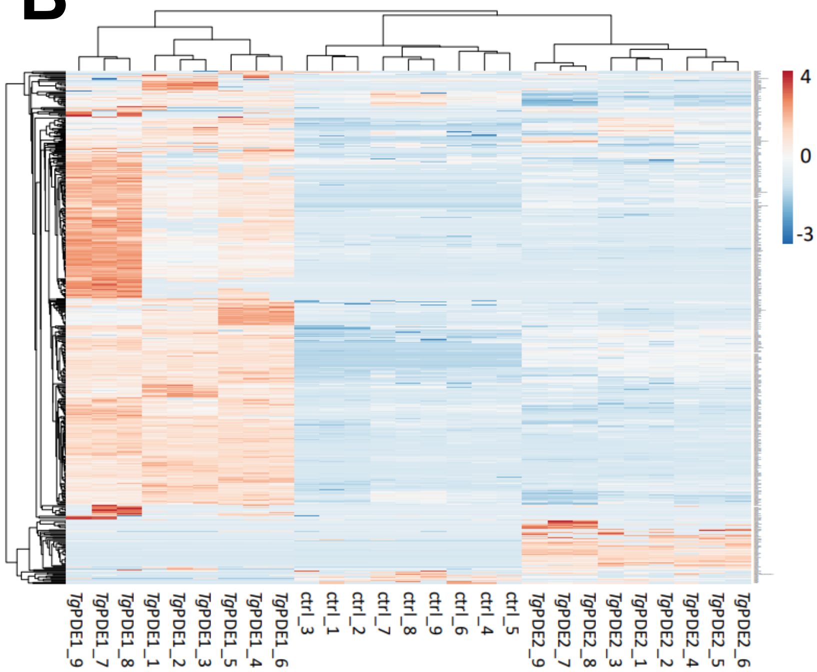

C

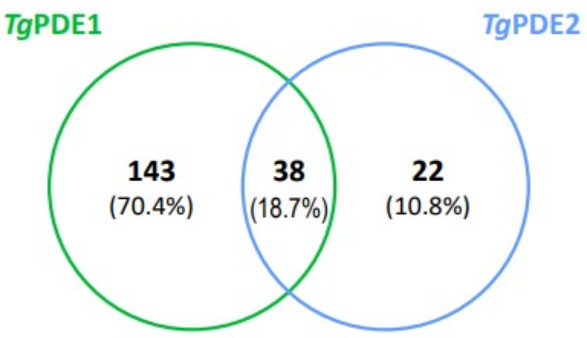

D

*Tg*PDE1	*Tg*PDE2	*Tg*PDE1 and *Tg*PDE2
Phosphodiesterase (TGGT1_293000) (*Tg*PDE1)	Phosphodiesterase (TGGT1_202540) (*Tg*PDE2)	Serine/threonine specific protein phosphatase (TGGT1_264660)
Serine/threonine-protein phosphatase (TGGT1_271050)	Serine/threonine-protein phosphatase (TGGT1_251850)	CMGC kinase, CK2 family (TGGT1_223390)
Protein disulfide isomerase (TGGT1_208830)		Putative TCP-1 Chaperonin (TGGT1_318410)
Acid phosphatase (TGGT1_219320)		
Chaperonin protein BiP (TGGT1_311720)		
Casein kinase II subunit beta (TGGT1_268200)		
